# Cryptic diversity of the subfamily Calaphidinae (Hemiptera: Aphididae) revealed by comprehensive DNA barcoding

**DOI:** 10.1371/journal.pone.0176582

**Published:** 2017-04-27

**Authors:** Yerim Lee, Wonhoon Lee, Mariusz Kanturski, Robert G. Foottit, Shin-Ichi Akimoto, Seunghwan Lee

**Affiliations:** 1 Laboratory of Insect Biosystematics, Department of Agricultural Biotechnology, Seoul National University, Seoul, Republic of Korea; 2 Research Institute of Agriculture and Life Sciences, Seoul National University, Seoul, Republic of Korea; 3 Department of Plant Medicine and Institute of Agriculture & Life Science, Gyeongsang National University, Jinju, Korea; 4 Department of Zoology, Faculty of Biology and Environmental Protection, University of Silesia, Bankowa, Katowice, Poland; 5 Invertebrate Biodiversity, National Environmental Health Program, Agriculture and Agri-Food Canada, Ottawa, Ontario, Canada; 6 Laboratory of Systematic Entomology, Department of Ecology and Systematics, Graduate School of Agriculture, Hokkaido University, Kita-ku, Sapporo, Japan; Sichuan University, CHINA

## Abstract

Aphids are a species rich group comprising many important pests. However, species identification can be very difficult for aphids due to their morphological ambiguity. DNA barcoding has been widely adopted for rapid and reliable species identification as well as cryptic species detection. In this study, we investigated cryptic diversity in the subfamily Calaphidinae (Hemiptera: Aphididae) based on 899 sequences of *cytochrome c oxidase I* (*COI*) for 115 morphospecies (78 species collected in this study and sequences of 73 species downloaded from Genbank). Among these 115 morphospecies, DNA barcoding results of 90 (78.3%) species were identical to results of morphological identification. However, 25 (21.7%) morphospecies showed discrepancies between DNA barcoding and traditional taxonomy. Among these 25 discordances, a total of 15 cryptic species were identified from 12 morphospecies. We also found three morphologically distinct species pairs that sharing DNA barcoding. Based on molecular operational taxonomic unit (MOTU) estimation, we discussed on species delimitation threshold value for these taxa. Our findings confirm that Calaphidinae has high cryptic diversity even though aphids are relatively well-studied.

## Introduction

Detecting cryptic species is essential for precise species diversity estimation [1]. With the availability of DNA barcoding methods, recognition of cryptic species has been increased over the past decades [1]. To date, DNA sequences of approximately 10% of all described species (ca. 160,000) are deposited in open access databases such as Barcode of Life Data Systems (BOLD) and the National Center for Biotechnology Information (NCBI) [2–3]. More recently, some researchers have suggested that DNA-sequenced-based taxonomic description alone is not only enough for species identification but also can be used as an alternative to classical taxonomy [4]. Indeed, DNA barcoding has been contributed to resolve morphological ambiguity in various taxa [5–9] with identification accuracy rate of 97% [10–13]. Therefore, DNA barcoding enables the detection of cryptic species and reassessment of species diversity.

Aphids are small and soft-bodied plant sap sucking insects. Over 5,000 of aphid species have been described worldwide [14], including many important pests in agriculture, forestry, and species of quarantine importance [15–19]. However, the lack of taxonomically informative characters and morphological plasticity of aphids make species recognition challenging [20]. Notably, in the complex life cycle of aphids, multiple morphs within one species can occur due to seasonal changes [21]. Numerous biotic factors such as host-plant relationships [22–23], natural enemies [24], ant attendance [25], maternal effects [26], endosymbionts [27], and infectious microorganisms [28] can affect intraspecific plasticity of aphids. Various abiotic factors, such as climate, temperature and photoperiod [29] also have effects on their intraspecific plasticity. Conversely, extremely similar or even morphologically indistinguishable species can occur among aphids [30–32]. The utility of DNA barcoding for aphid species identification has been demonstrated at family level [7, 13, 30]. It is also useful for some subfamilies such as Eriosomatinae [33], Greenideinae [34], and Lachninae [35]. According to these studies, DNA barcoding can provide rapid and reliable identification results for aphids. However, no assessment has been attempted for the subfamily Calaphidinae.

The subfamily Calaphidinae (Hemiptera: Aphididae) is the second largest subfamily in family Aphididae. About 398 valid species belonging to 59 genera have been described in the world [14, 36–37]. Most calaphidine aphids feed on woody angiosperms belonging to 16 plant families such as Betulaceae, Fagaceae, Juglandaceae, Lythraceae, Myricaceae, and Ulmaceae although some species feed on herbaceous plants belonging to Fabaceae and Poaceae [37–38]. Many aphid species belonging to this subfamily are economically important pests, causing injury and transmitting viral diseases to cultivated plants such as leguminous crops, fruit, and landscape trees [39–40]. For example, *Therioaphis trifolii* (Monell, 1882), *Melanocallis caryefoliae* (Davis, 1910) and *Monellia caryella* (Fitch, 1855) are notorious aphid pest have caused large agricultural economic losses [41–43]. Some aphid pest, such as *Sarucallis kahawaluokalani* (Kirkaldy, 1907), *Shivaphis celti* Das, 1918 and *Tinocallis* spp. have been dispersed from their geographic origins to different continents [18, 44–46]. However, assessing calaphidine species can be difficult and time consuming since their considerable morphological variation based on seasonal changes and various biotic factors [25, 37]. The application of DNA barcoding would assist in rapid and accurate identification of species in this subfamily. It can also aid the detection of cryptic diversity.

In this study, we provided the first comprehensive assessment of DNA barcodes for the subfamily Calaphidinae. A total of 501 *Cytochrome oxidase I* (*COI*) sequences of 78 morphospecies collected in Korea and other countries from 2001 to 2015 were analyzed. The objectives of this study were i) to clarify delimiting species boundaries in morphologically ambiguous taxa, ii) to test the effectiveness of DNA barcoding in this taxa, and ultimately iii) to detect hidden species diversity.

## Materials and methods

### Ethics statement

No permission was required for sampling at the sites studied. No endangered or protected species are included in this study.

### Taxon sampling

A total of 501 aphid individuals of 78 species were collected in Asia: Korea (382 specimens of 52 species), China (20 specimens of 8 species), Japan (23 specimens of 8 species) and Laos (2 specimens of 1 species); Europe: Czech Republic (15 specimens of 7 species), Poland (16 specimens of 7 species) and UK (5 specimens of 1 species); North America: USA (29 specimens of 14 species) and Oceania: New Zealand (9 specimens of 4 species) from 2001 to 2015 (S1 Table). Each specimen was preserved in 95–99% ethanol at -20°C for genomic DNA extraction.

### Species identification

501 individuals were mounted in Canada balsam following the method of Blackman & Eastop [16] and Martin [47]. Measurements for each specimen were taken from digital images by using image analysis software (Active measure ver. 3.0.3 from Mitani Co. Ltd, Japan). Digital images were taken by a digital camera attached to a microscope (Leica 400B, Leica Microsystems, Germany). All slide specimens were deposited in the College of Agriculture and Life sciences, Seoul National University (CALS SNU), the Republic of Korea.

### DNA extraction and DNA barcoding

Genomic DNA was extracted from each sample selected from each colony by using the DNeasy Blood & Tissue kit (Qiagen, Dusseldorf, Germany) according to the modified manufacturer’s protocols. To confirm morphological features, we used a nondestructive method: each whole-bodied specimen was put into a mixture of 90μl of ATL buffer and 10 μl of proteinase K incubated without pulverization. After 24 h incubation, 90μl AL buffer was added and incubated for another 10 min. The solution was gently pipetted into a mini spin column leaving the cuticle of the specimen which was slide mounted.

A 658 bp of *COI* gene region, generally called as ‘barcoding region’ was amplified using a universal primer set: LCO1490 5’-GGTCAACAAATCATAAAGATATTGG-3’ and HCO2198 5’-TAAACTTCAGGGTGACCAAAAAATCA-3’ [48]. Polymerase chain reaction (PCR) was conducted with AccuPower PCR PreMix (Bioneer, Daejeon, Korea) in 20 ml reaction mixtures under the following conditions: initial denaturation at 94°C for 3 min; followed by 35 cycles at 94°C for 30s, an annealing temperature of 45.2°C for 30s, an extension at 72°C for 1min; and the final extension at 72°C for 5min. All PCR products were assessed 1.5% agarose gel electrophoresis. Successfully amplified samples were purified using a QIAquick PCR purification kit (Qiagen, Inc.), and then sequenced directly using an automated sequencer (ABI PrismH 3730 XL DNA Analyzer) at Macrogen Inc. (Seoul, Korea).

### Molecular analyses

All sequences to be analyzed were initially assembled and examined using Seqman pro ver. 7.1.0 (DNA star, Inc., Madison, Wisconsin, USA). Poor quality sequences with ambiguous peaks were removed. We used the molecular identification criteria of putative orthologues and paralogues according to Moulton et al. [49] and Fontaneto et al. [50], to prevent misleading by nuclear mitochondrial pseudogenes (Numts) and heteroplasmy. A total of 501 *COI* sequences of 78 species including previously unknown sequences of 42 species were newly generated for the molecular analyses. Additionally, 398 *COI* sequences of 73 species were downloaded from Genbank using keyword ‘*COI*’ and ‘Calaphidinae’ (S2 Table). As a result, the final dataset consisted of 899 sequences of 115 species (S1 and S2 Tables).

These data was aligned using online utility MAFFT ver. 7 alignment package [51] and MEGA 6 [52]. In this step, we removed uncertain anterior and posterior regions were removed. Fnally, ≥ 546 bp was used for analyses. For the aligned dataset, a neighbor-joining analysis was conducted using MEGA 6 based on Kimura-2-Parameter (K2P) model [53], the best for species level analysis, particularly for those with low distances [54]. Intra- and inter-specific distances in different taxonomic levels were calculated using pairwise distance method based on the K2P model [53] using MEGA 6.

To infer species delimitation criteria based on a partial *COI* gene in this subfamily, we performed molecular operational taxonomic units (MOTUs) estimation by using two effective tools to delimit molecular species. First, Automatic Barcode Gap Discovery (ABGD) analysis was conducted to automatically delimit sequences into hypothetical molecular species [55] (http://wwwabi.snv.jussieu.fr/public/abgd) by contrasting inter- and intra-specific distances. Standard settings were used with two values of relative gap width (*X* = 1 and *X* = 1.5) based on Kimura K80 model. Additionally, Bayesian Poisson Tree Processes (bPTP) analysis as implemented on the Exelixis Lab web-server (http://species.h-its.org/ptp/) was performed. This method delimits species based on the phylogenetic species concept [56]. Compared to the generalized mixed Yule coalescent (GMYC) model, bPTP model is a more robust and simpler method [57]. The required rooted phylogenetic input tree was drawn using RAxML [58] with GTR+G+I substitution model.

## Results

### Genetic variation of morphospecies

A total of 501 *COI* sequences (≥ 546 bp) from 78 morphospecies belonging to 36 genera of four subtribes, Calaphidina, Monaphidina, Myzocallidina and Panaphidina were newly generated in this study (S1 Table). All sequences are deposited in Genbank (KY306805-KY307305). Results of genetic divergence at different taxonomic levels are summarized in Table 1.

**Table 1:** Genetic divergences in different taxonomic level within Calaphdinae.

Comparison within	Mean (%)	Minimum (%)	Maximum (%)
Species	0.7	0	16.6
Genus	21.1	0	9.5
Subtribe	13.4	3.4	22.8
Tribe	13.8	3.4	23.1

The overall mean distance was 13.2% for the final dataset of 899 sequences of 115 species. The mean interspecific distance ranged from 4.19% to 13.3% at genus level. The mean genetic distance between genera ranged from 11.92% to 14.51% at subtribe level.

**Fig 1:**
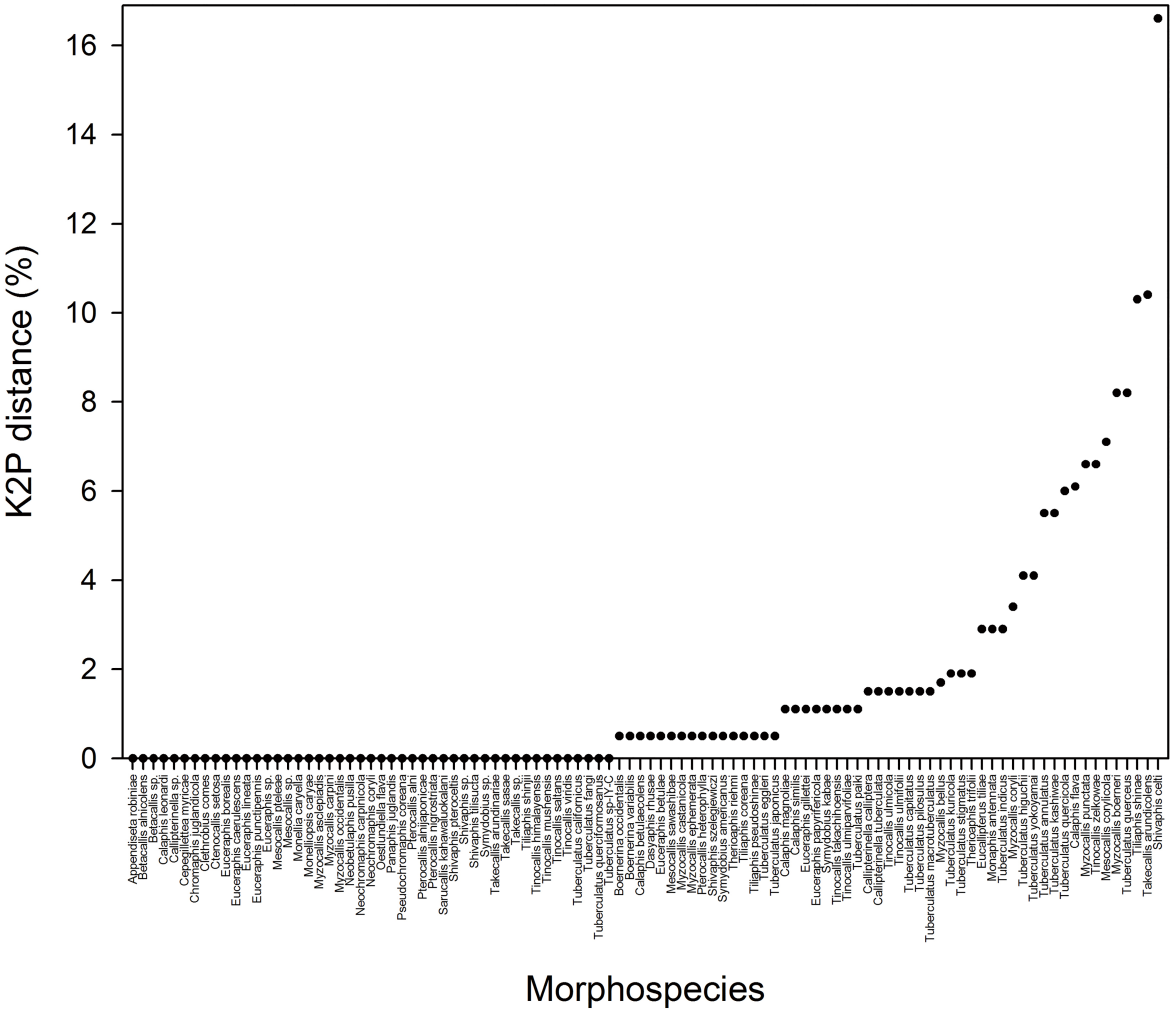
Maximum intraspecific distances (%) based on Kimura-2-parameter (K2P) for 100 morphospecies.

Intraspecific genetic distance was calculated for the 100 of 115 morphospecies. For the remaining 15 species, intraspecific distances could not be calculated because there was only one individual representing each species. The mean intraspecific distance ranged from 0 to 6.0% in each species. Among these 100 morphospecies, 78 species showed very low to moderate genetic divergences (below 1.5%, Fig 1). Another 4 species showed ambiguous intraspecific distances ranging from 1.7% to 1.9% (Fig 1). However, the remaining 18 species showed relatively high intraspecific distances ranging from 2.9% to 16.6% (Fig 1, Table 2). Such a high level of intraspecific distance indicate that there might be potential cryptic species and/or misidentified sequences in these of 18 species (Table 2).

**Table 2:** 19 Cases of having high intraspecific distances.

Subtribe	Species	No. of subgroups	Max. Intraspecific distance
Calaphidina	*Calaphis flava*	3	6.1%
Monaphidina	*Monaphis antennata*	2	2.9%
Myzocallidina	*Myzocallis boerneri*	2	8.2%
	*Myzocallis coryli*	2	3.4%
	*Tuberculatus annulatus*	2	5.5%
	*Tuberculatus higuchii*	4	4.1%
	*Tuberculatus indicus*	2	2.9%
	*Tuberculatus kashiwae*	2	5.5%
	*Tuberculatus punctata*	2	6.6%
	*Tuberculatus querceus*	2	8.2%
	*Tuberculatus quercicola*	2	6.0%
	*Tuberculatus yokoyamai*	2	4.1%
Panaphidina	*Eucallipterus tiliae*	2	2.9%
	*Mesocallis corylicola*	2	7.1%
	*Shivaphis celti*	2	16.6%
	*Takecallis arundicolens*	3	10.4%
	*Therioaphis trifolli*	2	1.9%
	*Tiliaphis shinae*	2	10.3%
	*Tinocallis zelkowae*	2	6.6%

### MOTUs estimation

**Fig 2:**
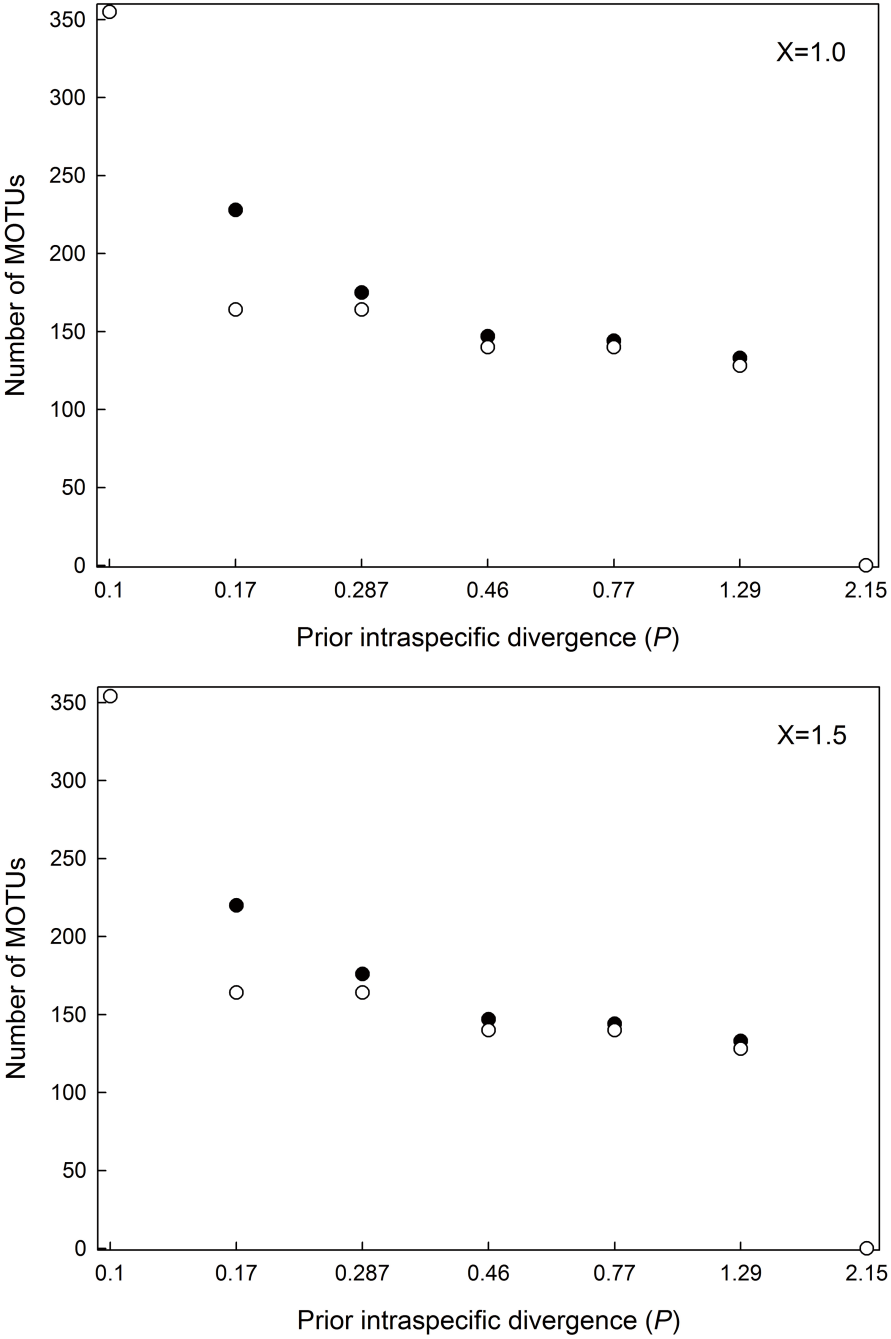
Number of MOTUs by the prior intraspecific divergence using ABGD with two values of relative gap width. (A) *X* = 1. (B) *X* = 1.5.

**Fig 3:**
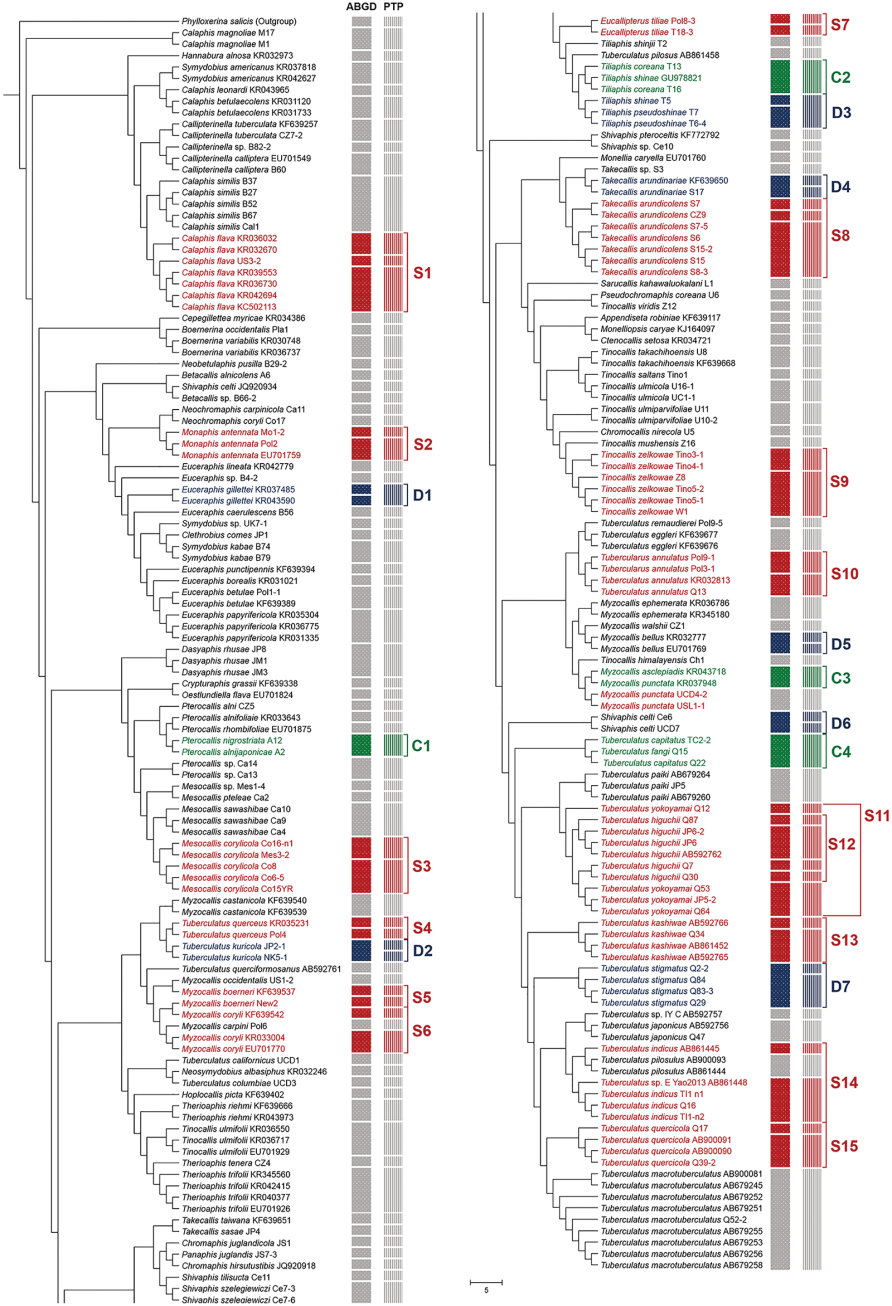
Maximum likelihood *COI* gene tree with deliminated MOTUs by ABGD and PTP analyses. (Red S) Subdivided morphospecis. (Green C) combined morphologically different species. (Blue D) discordant between ABGD and PTP results.

The number of MOTUs determined by ABGD differed slightly depending on the value of the relative gap width (Fig 2). When *p* value was set at 0.0129, the number of MOTUs was 133 at relative gap width *X* = 1 or *X* = 1.5 (Fig 2). Based on ABGD result, 16 species were divided into 2–4 inner-groups in each species group (Fig 3). Three morphologically distinct species pairs: i) *Pterocallis alnijaponicae* and *P*. *nigrostriata*, ii) *Tiliaphis pseudoshinae* and *T*. *shinae* and iii) *Tuberculatus* (*Orientuberculoides*) *capitatus* and *T*. (*O*.) *fangi* were clustered together as a single MOTU, respectively (Table 3).

**Table 3:** Case of sharing low genetic distances between morphologically distinct species pairs.

Subtribe	Species 1	Species 2	Genetic distance
Myzocallidina	*Tuberculatus capitatus*	*Tuberculatus fangi*	0.5–1.3%
Panaphidina	*Pterocallis alnijaponicae*	*Pterocallis nigrostriata*	0.5%
	*Tiliaphis pseudoshinae*	*Tiliaphis shinae*	1.7–1.9%

The bPTP model recognized a total of 136 MOTUs except for outgroup based on the maximum likelihood method. Among 115 morphospecies, 19 species were subdivided into 2–4 inner-groups in each species group (Fig 3). This result was similar not only based on the number of MOTUs, but also based on MOTU compositions obtained from ABGD (Fig 3). Overall, the bPTP model tended to be more sensitive to MOTU delimitation. ABGD and bPTP resulted in different estimates for 8 morphospecies (Fig 3). For example, 4 morphospecies, *Takecallis arundinariae*, *Tuberculatus* (*Nippocallis*) *kuricola*, *Tuberculatus* (*Arakawana*) *stigmatus* and *Myzocallis* (*Lineomyzocallis*) *bellus*, were subdivided into two groups each in the bPTP results. However, no subdivision was detected for these 4 species based on ABGD results (Fig 3). On the contrary, *Tiliaphis shinae* and *T*. *pseudoshinae* pair was clustered together as a single MOTU in the bPTP model (Fig 3).

### Neighbor joining analysis: Case of species delimitation

The Neighbor joining tree (NJ tree) was derived for these 899 *COI* sequences of the 115 species (S1 Fig). For majority of cases, sequence clusters in the NJ tree showed high congruence with morphological identification results. Practically, 90 species (78.3%) of all species could be clearly identified by *COI* sequence. However, for the remaining 25 species (21.7%), discrepancies between morphology and barcode based identification were detected. DNA barcoding analyses revealed the following: i) 15 cryptic species from 12 morphospecies, ii) six possible cryptic or potential misidentified sequences in the Genbank, iii) three morphologically distinct species pairs that sharing a single MOTU, and iv) four species with ambiguous inter- and intra-specific distances. Detailed results for these four cases are described as follows.

#### Case I: Discovering cryptic species

A total of 15 cryptic species from 12 morphospecies were found based on original description and other information of each morphospecies such as host-plant association and distributional information. Because in any case, it is impossible to identify DNA barcode for type materials. For each case, comparison between original and cryptic species was discussed.

**Fig 4:**
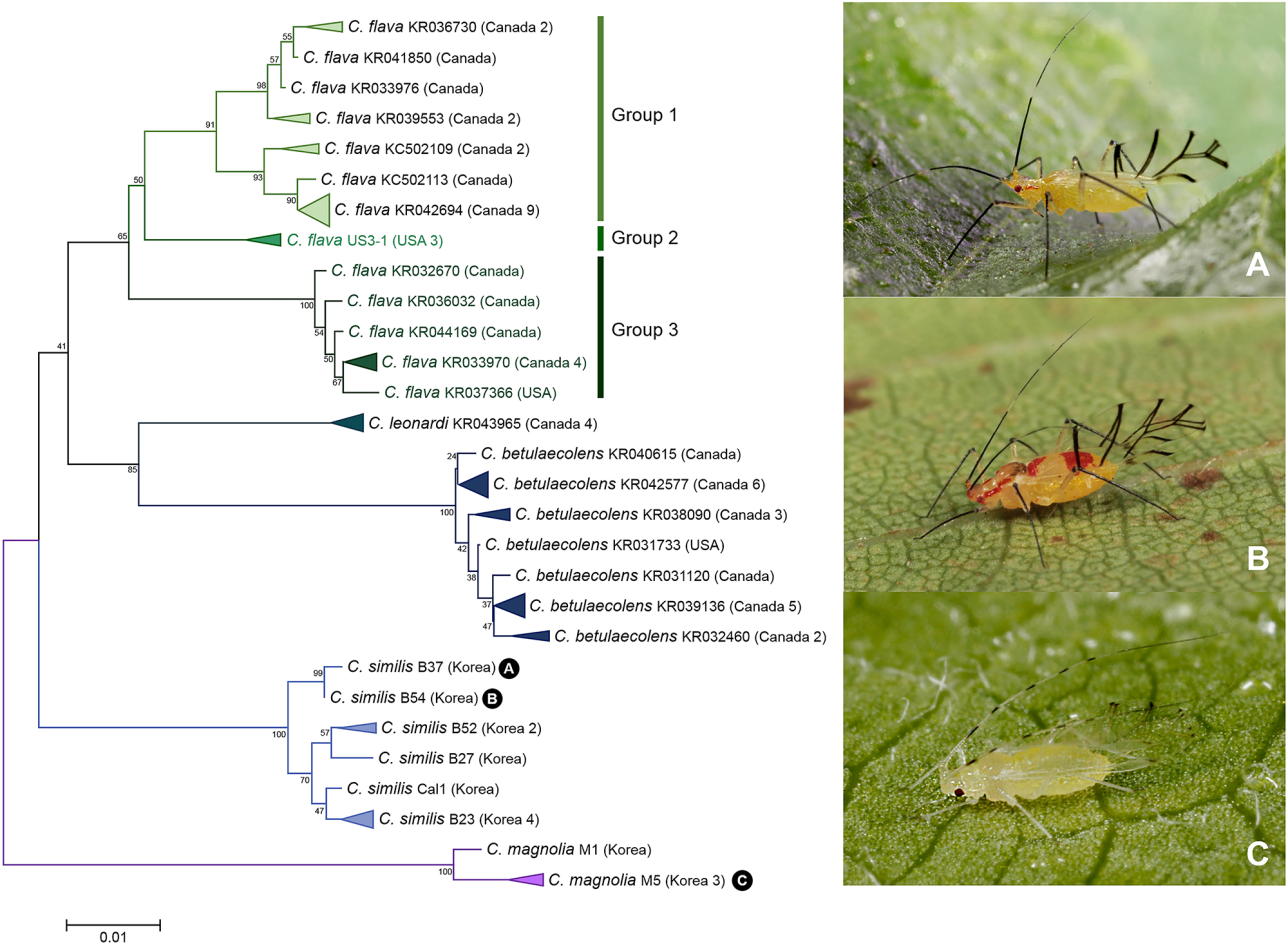
Neighbor-joining tree of *COI* partial gene sequences of *Calaphis* spp. (66 sequences of 5 morphospecies).

A total of 66 individuals of 5 species belonging to genus *Calaphis* were analyzed in this study. DNA barcoding detected 2 cryptic species in *Calaphis flava* (Fig 4). Between group 1 and group 2, intergroup divergence was 2.9–4.0% (Fig 4). Group 3 consisted of 7 Canadian and 1 American individual was distinct from group 1 and group 2 with 4.5–6.1% of genetic divergence (Fig 4). This species was originally distributed throughout Europe and East Asia. Now it is regarded as a widespread species found in South Africa, Australia and North America. Thus, further studies are needed to compare European individuals of *C*. *flava* in the future study to investigate whether this species is a real cosmopolitan species or a species complex.

**Fig 5:**
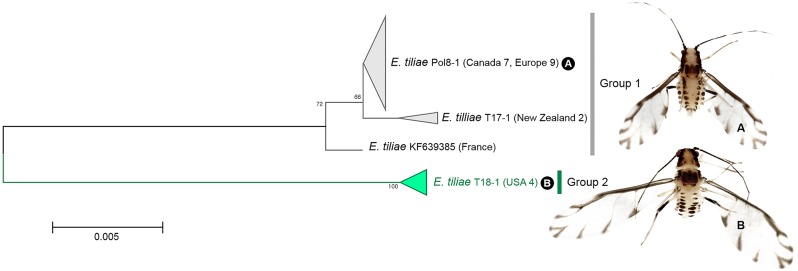
Neighbor-joining tree of *COI* partial gene sequences of *Eucallipterus* spp. (23 sequences).

A total of 23 individuals of *Eucallipterus tiliae* were collected from Europe: Czech Republic, France, Italy and Poland, North America: Canada and USA, and Oceania: New Zealand. ABGD, bPTP (Fig 3) and NJ tree revealed 1 cryptic species (group 2) of *E*. *tiliae* with about 2.9% of intergroup genetic divergence (Fig 5). Morphologically, individuals in group 1 and 2 are very similar. However, cryptic species (group 2) is distinguishable from typical *E*. *tiliae* (group 1) by having longer length of 3rd–5th antennal segment and 2nd tarsal segment (S2 Fig). In genus *Eucallipterus*, only two species have been described in the world. *E*. *tiliae* is a common species widely distributed throughout Europe across central Asia, and South-Africa. It has also been introduced into North America and New Zealand [37]. Our results suggest that this species might be a species complex rather than a cosmopolitan species.

**Fig 6:**
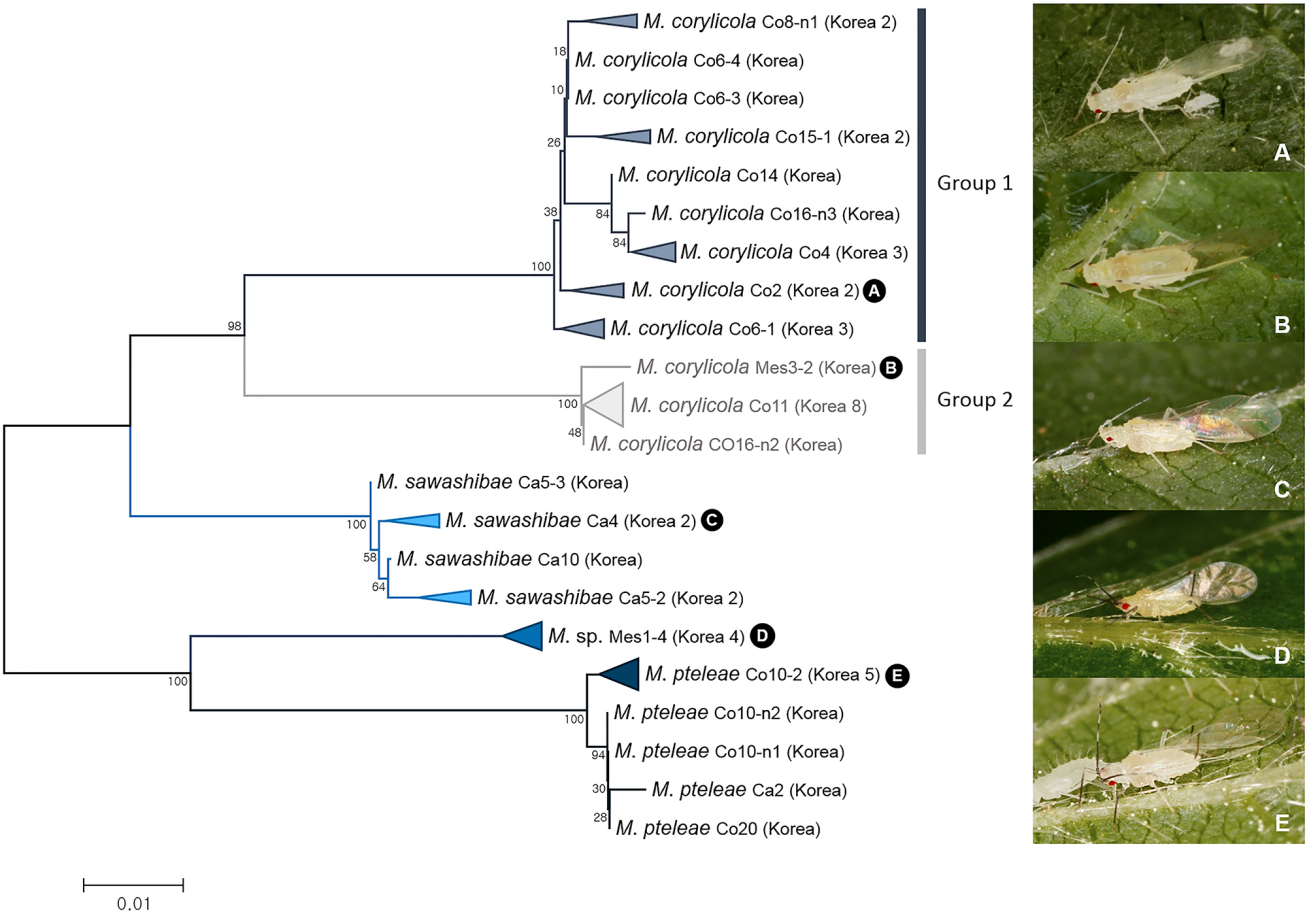
Neighbor-joining tree of *COI* partial gene sequences of *Mesocallis* spp. (45 sequences of 4 morphospecies).

ABGD and bPTP analyses on 45 individuals of four Korean species of *Mesocallis* disclosed one cryptic species in *M*. *corylicola* (Figs 3 and 6). *M*. *corylicola* (group 1) and cryptic species (group 2) showed 5.5–7.1% of genetic distance (Fig 6). In contrast to such a high level of genetic distance, the cryptic species and *M*. *corylicola* are superficially similar in morphology. Compared to *M*. *corylicola*, the cryptic species has a shorter siphunculi and longer length of ultimate rostral segment (S3 Fig). Host-plant preference appeared to differ between the two species. Most *M*. *corylicola* was collected on *Corylus sieboldiana* while most cryptic species were collected on *C*. *heterophylla* (S1 Table). According to the original description, original species was collected on *Corylus sieboldiana* [59]. Thus, the two groups have different *COI* sequences, host-plant preference, and morphology.

**Fig 7:**
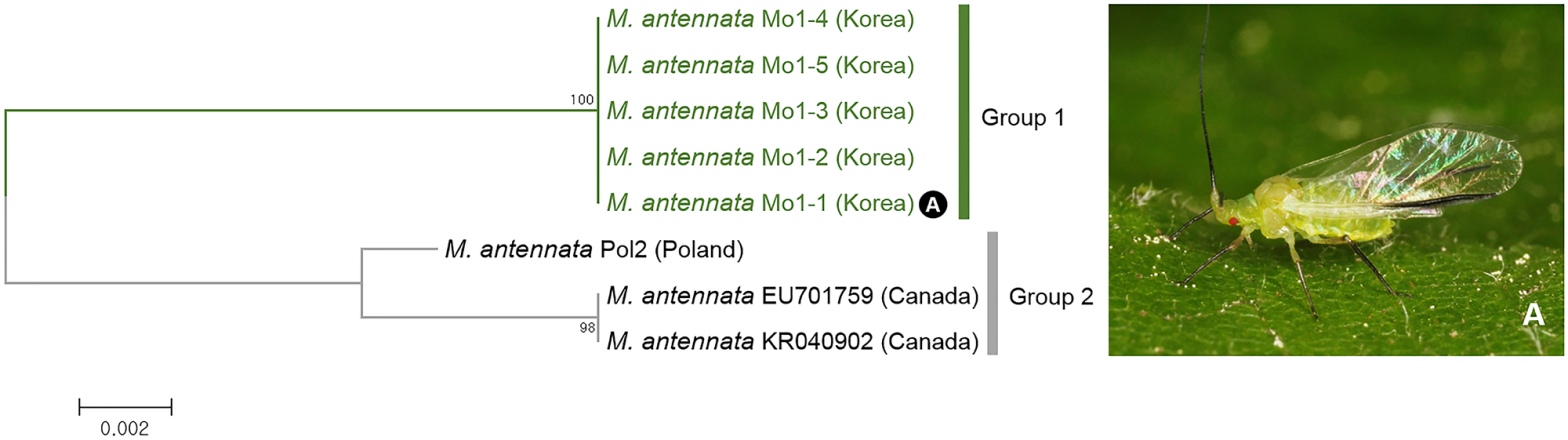
Neighbor-joining tree of *COI* partial gene sequences of *Monaphis antennata* (8 sequences).

Eight individuals of *Monaphis antennata* collected from Canada, Korea, and Poland were analyzed with ABGD, bPTP (Fig 3), and NJ tree (Fig 7). Korean individuals formed a group (group 1) distinct from Polish and Canadian individuals with 2.4–2.9% intergroup genetic divergence (Fig 7). *Monaphis* is a monotypic genus originally described from Europe. This species lives solitarily on *Betula* spp. In Korea, *Monaphis* is extremely rare. It has only been collected on *Betula schmidtii*. In Europe and Japan, it has been collected on *B*. *pendula* [60–61], *B*. *maximowicziana* and *B*. *platyphylla* var. *japonica* [59]. Although we could not perform morphological comparisons on subgroups, the Korean cluster seems to be a distinct species based on the molecular divergence level.

**Fig 8:**
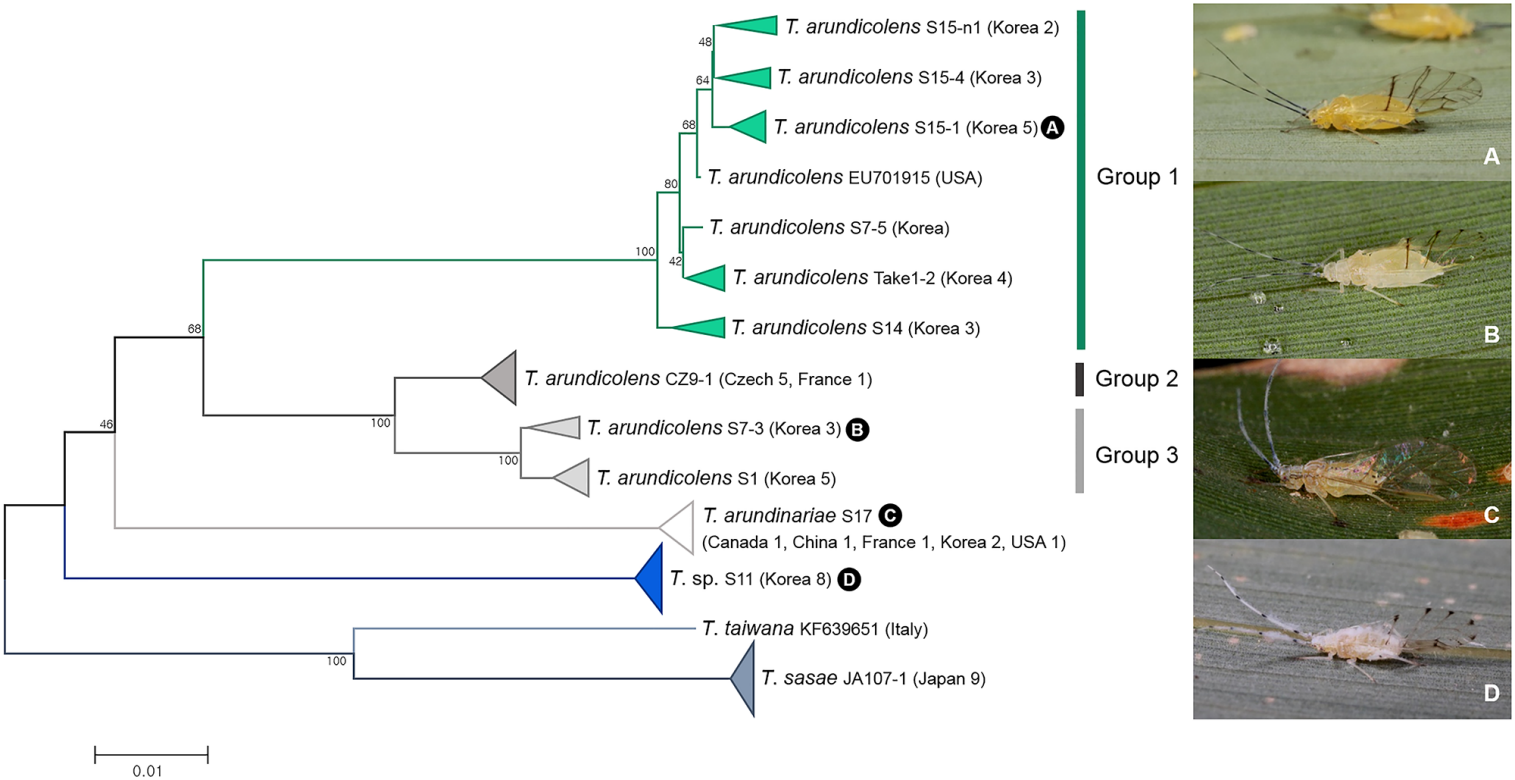
Neighbor-joining tree of *COI* partial gene sequences of *Takecallis* spp. (58 sequences of 5 morphospecies).

We analyzed 58 individuals of five *Takecallis* spp. collected from East Asia: China, Japan, and Korea, Europe: Czech Republic, France, and Italy, and North America: Canada and USA. According to both ABGD and bPTP analyses, *T*. *arundicolens* were separated into three groups (Figs 3 and 8). Genetic divergence between group 1 and group 2+3 ranged from 7.6% to 10.4%. Genetic divergence between group 2 and group 3 was 2.5%. In each group, morphological differences were only detected between alatoid nymphs. Alatoid nymphs in group 1 could be distinguished by a dark colored cauda with short dorsal abdominal setae (S4 Fig). Group 2 and group 3 shared similar morphology. However, group 3 could be distinguished from group 2 by shorter siphunculi with long filiform setae on the body (S6 Fig 4). *T*. *arundicolens* is one of common bamboo feeding species. Originally, this species was described from East Asia. It has been introduced into Europe and North America. Our results indicate that European populations (group 2) might be species distinct from Asian species (group 1 and group 3).

**Fig 9:**
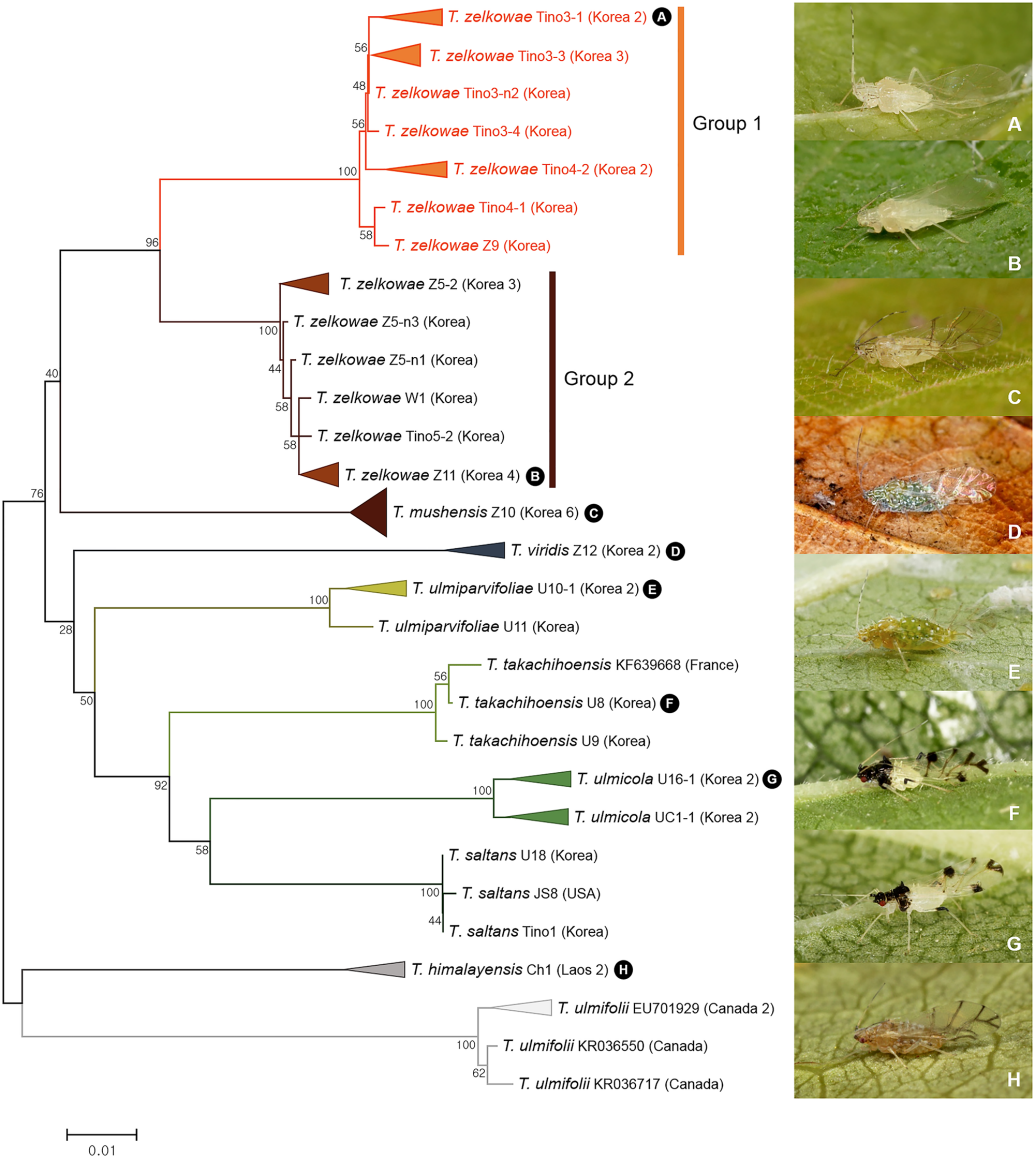
Neighbor-joining tree of *COI* partial gene sequences of *Tinocallis* spp. (48 sequences of 9 morphospecies).

A total of 48 individuals of nine *Tinocallis* spp. collected from East Asia: Korea and North America: Canada and USA were analyzed. Both ABGD and bPTP analyses recognized two subgroups (group 1 and group 2) among 22 individuals of *T*. *zelkowae* collected from Korea (Fig 3). These groups showed genetic divergence of 5.5–6.5% (Fig 9). Morphologically, individuals of group 1 have longer ultimate rostral segments than those of group 2 with pigmented dorsal abdominal elevations (S5 Fig). Group 1 is relatively rare. It was only collected on native *Zelkova serrata* (var. *latifolia*) growing in Korean mountain areas. *T*. *zelkowae* of the group 2 is one of the most common species dwelling on *Zelkova* trees in urban area (mostly the Japanese species *Zelkova serrata* (var. *japonica*)). Results of this study indicate that group 1 might be species separated from group 2 with different host-plant association.

**Fig 10:**
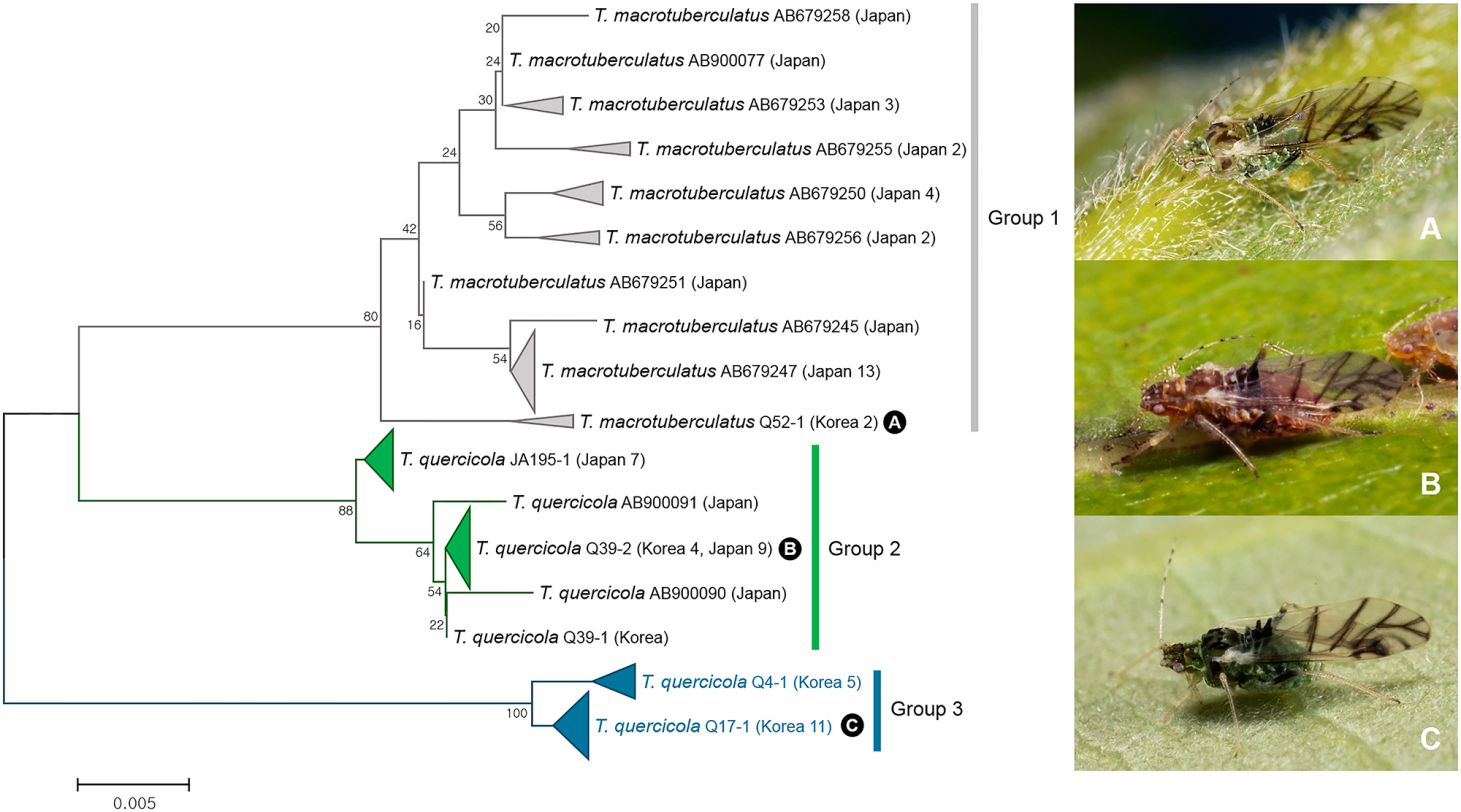
Neighbor-joining tree of *COI* partial gene sequences of subgenus *Acanthocallis* spp. (69 sequences of 2 morphospecies).

In genus *Tuberculatus*, a total of seven cryptic species were discovered. Detailed results for each subgenus are provided as follows. A total of 69 individuals of two species belonging to the subgenus *Acanthocallis* collected from Japan and Korea were analyzed. Of 21 Korean individuals of *Tuberculatus* (*Acanthocallis*) *quercicola*, 16 individuals formed group 3, distinct from Japanese and remaining 5 Korean individuals of *T*. (*A*.) *quercicola* (group 2) with about 5.2% of intergroup genetic divergence (Figs 3 and 10). Individuals of group 3 were different from group 2 only by having shorter setae on 3rd antennal segment with more setae on 4th–5th antennal segments (S6 Fig). Watanabe et al. [62] have reported that *Acanthocallis* species tend to have high host specificity. It has been shown that Japanese *T*. (*A*.) *quercicola* and *T*. (*A*.) *macrotuberculatus* have distinct host-plant associations with *Quercus mongolica* spp. *crispula* and *Q*. *dentate*, respectively [62]. Likewise, three Korean species: *T*. (*A*.) *quercicola* in group 2, *T*. (*A*.) *macrotuberculatus* in group 1, and cryptic species in group 3 showed distinct host-plant association with *Q*. *mongolica*, *Q*. *dentate*, and *Q*. *aliena*, respectively. Therefore, different host associations between species can be used for species identification in this group.

**Fig 11:**
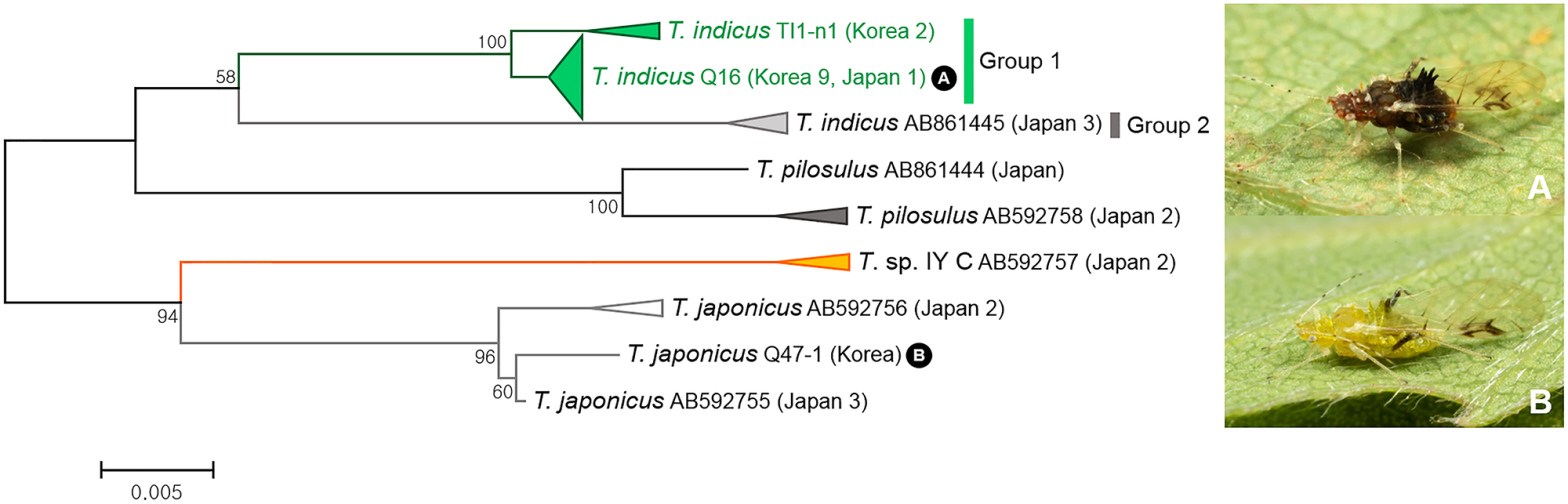
Neighbor-joining tree of *COI* partial gene sequences of subgenus *Acanthotuberculatus* spp. (26 sequences of 4 morphospecies).

A total of 26 individuals of four species belonging to subgenus *Acanthotuberculatus* were analyzed. As shown in Figs 3 and 11, 14 specimens of *Tuberculatus* (*Acanthotuberculatus*) *indicus* were split into two subgroups with intergroup genetic divergence ranging from 2.4% to 2.9%. Nine specimens collected from Korea were identical to undescribed species *Tuberculatus* sp. E (Yao, unpublished, *COI* sequence Genbank accession no. AB861448). *Tuberculatus* (*A*.) *japonicus* and undescribed species, *Tuberculatus* sp. IY-C [63], showed inter-specific distance of 4.5% to 5.1%. No morphological comparisons was undertaken.

**Fig 12:**
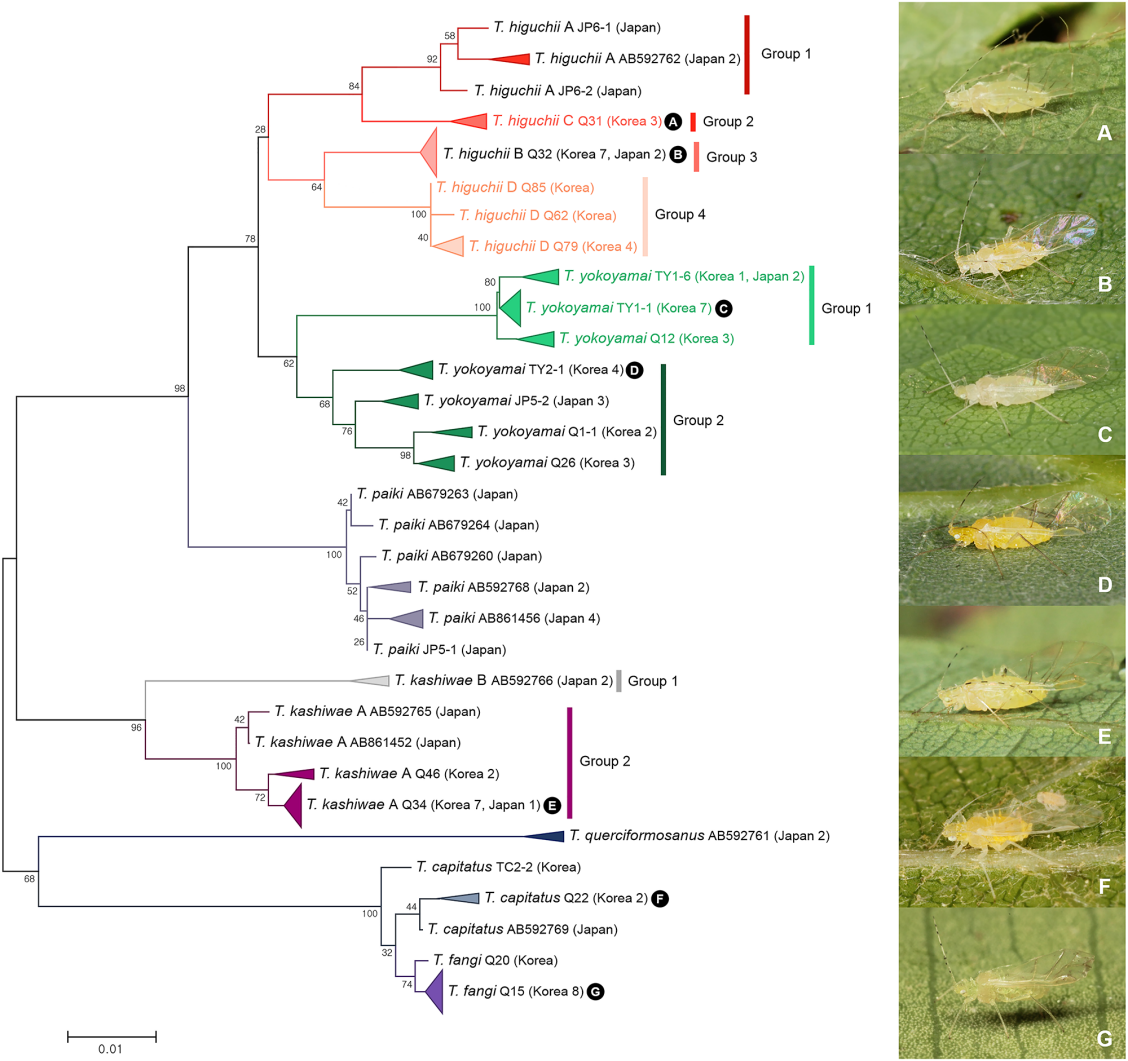
Neighbor-joining tree of *COI* partial gene sequences of subgenus *Orientuberculoides* spp. (86 sequences of 7 morphospecies).

DNA barcoding uncovered five cryptic species among 86 specimens of seven species in subgenus *Orientuberculoides* (Fig 3). *Tuberculatus* (*Orientuberculoides*) *higuchii* was subdivided into four subgroups, including previously detected species *T*. (*O*.) *higuchii* A (group 1) and *T*. (*O*.) *higuchii* B (group 3) [63] and two newly detected subgroups (Fig 12). For convenience, these newly detected subgroups were named as *T*. (*O*.) *higuchii* C (group 2) and *T*. (*O*.) *higuchii* D (group 4). Among those four subgroups, intergroup genetic divergence of 2.8% to 4.1% was observed (Fig 12). *T*. (*O*.) *higuchii* A has shorter 2nd–4th antennal segments in comparison with other subgroups (S7 Fig). *T*. (*O*.) *higuchii* B is distinct from other subgroups by having shorter siphunculi (S7 Fig). *T*. (*O*.) *higuchii* C has slightly longer second tarsal segments and cauda (S7 Fig). *T*. (*O*.) *higuchii* D is distinguished from others by having more secondary sensoria on the 3rd antennal segment (S7 Fig).

Fourteen specimens of *Tuberculatus* (*Orientuberculoides*) *kashiwae* collected from Japan and Korea were divided into two subgroups with intergroup genetic divergence of 5.1% to 5.5% (Fig 12), confirming earlier suggestion by Yao [63]. All Korean specimens were clustered together with *T*. (*O*.) *kashiwae* B [63]. Only two sequences from Japan formed group 1 (*T*. (*O*.) *kashiwae* A).

A total of 25 individuals of *Tuberculatus* (*Orientuberculoides*) *yokoyamai* collected from Japan and Korea were analyzed. Results are shown in Fig 12. ABGD and bPTP analyses detected two cryptic species within *T*. (*O*.) *yokoyamai*, showing genetic divergence of 3.5% to 4.1% (Fig 12). Thirteen specimens collected from Japan and Korea formed group 1 together with *Tuberculatus* sp. D Yao (unpublished, *COI* sequence Genbank accession no. AB861455). Morphologically, group 1 was distinguished from group 2 by having longer length of 5th antennal segment, siphunculi, and cauda (S8 Fig). There was no host-plant difference between the two groups.

**Fig 13:**
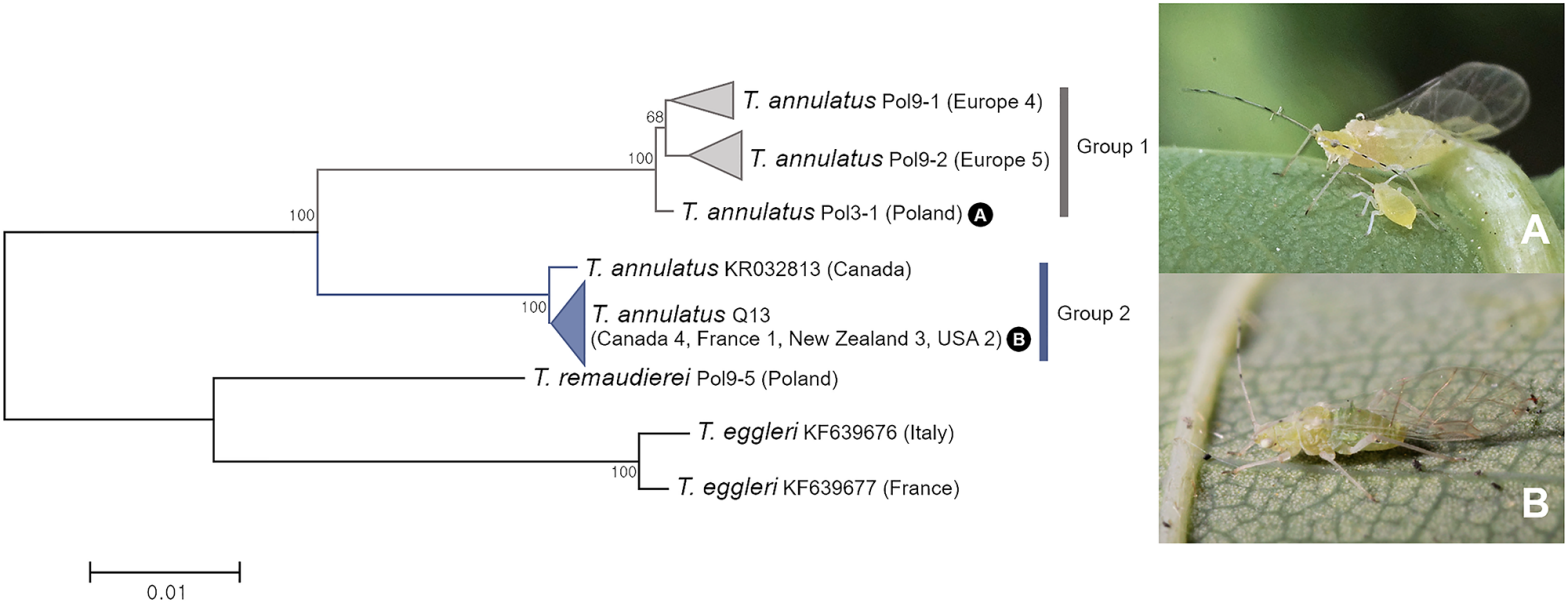
Neighbor-joining tree of *COI* partial gene sequences of subgenus *Tuberculoides* spp. (19 sequences of 3 morphospecies).

*COI* sequences of 19 specimens of three species in the subgenus *Tuberculoides* revealed two groups within *Tuberculatus* (*Tuberculoides*) *annulatus* (Figs 3 and 13) with genetic divergence of 5.1–5.5%. All 10 individuals of group 1 were collected from Europe: France, Poland and UK. Group 2 comprised of 7 North American specimens, 3 New Zealand specimens, and one French specimen. One French specimen in group 2 has been regarded as an outlier in a previous study of Coeur d’Acier et al. [64]. However, this haplotype was present in North America and New Zealand. Morphological features of the two subgroups (group 1 and group 2) of *T*. (*T*.) *annulatus* were compared. Group 2 is distinguishable from group 1 by shorter antennae and smaller cauda knob with longer setae (S9 Fig). Based on molecular and morphological differences, group 1 and group 2 might be distinct species. It has been widely assumed that *T*. (*T*.) *annulatus* is introduced into North America, South America, Australia, and New Zealand from Europe [38]. However, such assumption need to be examined in future studies.

#### Case II: Discovering possible cryptic or misidentified sequences in NCBI

A total of 398 *COI* sequences of 73 species were downloaded from Genbank and used in this study. The following statements are only suggestions since specimen morphology could not be examined in this study.

**Fig 14:**
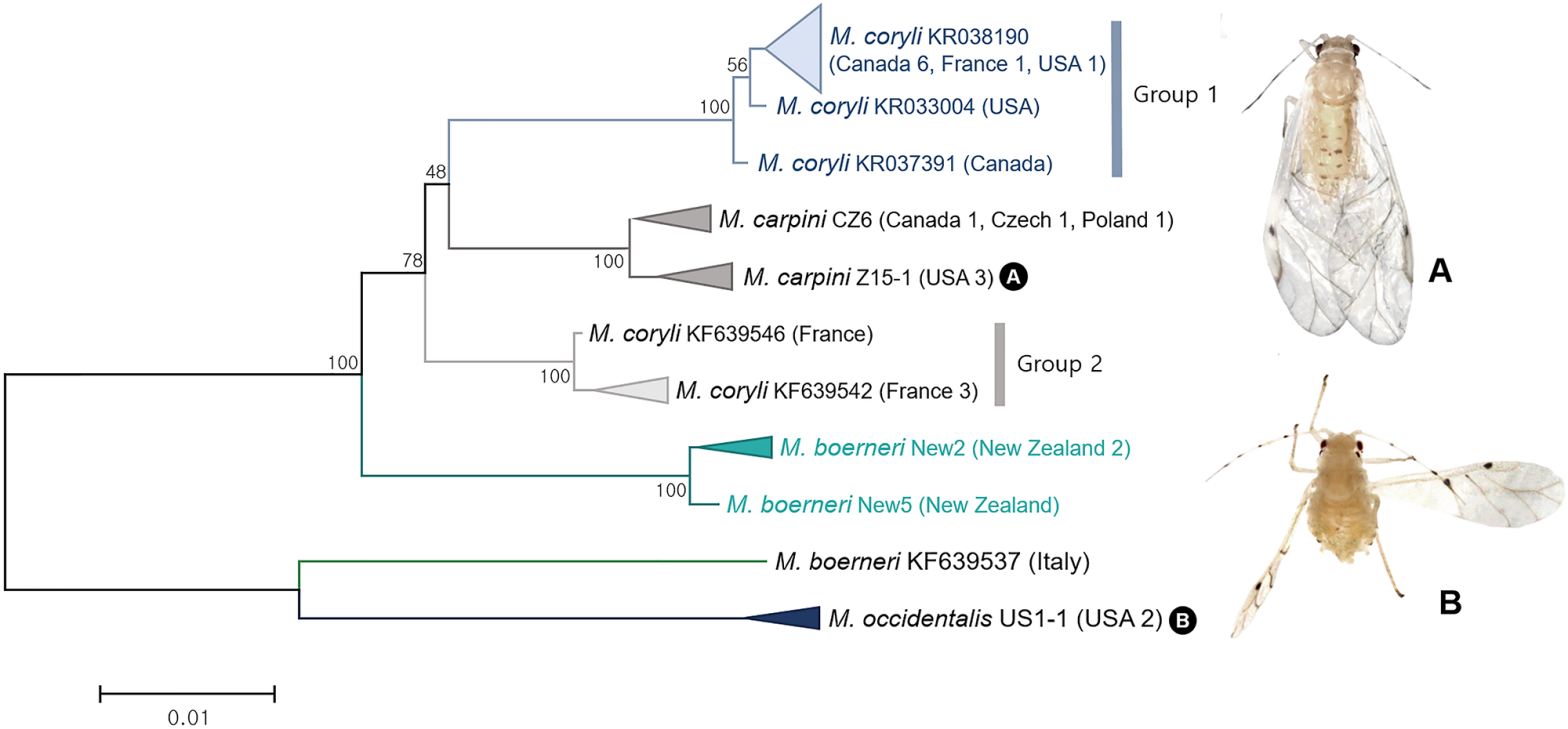
Neighbor-joining tree of *COI* partial gene sequences of subgenus *Myzocallis* spp. (26 sequences of 4 morphospecies).

In genus *Myzocallis*, a total of three possible discrepancies compared to current species concepts were identified. Detailed results of each subgenus are described as follows. We analyzed 26 sequences of four species belonging to subgenus *Myzocallis* collected from Europe: Czech Republic, France, Italy and Poland; North America: Canada and USA; and Oceania: New Zealand. ABGD and bPTP analyses revealed two subgroups in *Myzocallis* (*Myzocallis*) *coryli*. Group 1 and group 2 showed intergroup genetic divergence of 2.9–3.4% (Fig 14). Group 1 of *M*. (*M*.) *coryli* formed a sister group of *M*. (*M*.) *carpini* with genetic divergence of 2.7–3.1%. Group 1 mostly comprised of North American specimens except for one French specimen (Genabank accession no. KF639545) while all specimens of group 2 were from France. Coeur d’Acier et al. [64] have reported that *M*. (*M*.) *coryli* show exceptionally high intraspecific divergence due to the outlier. However, the outlier was the most common haplotype of *M*. (*M*.) *coryli* in our dataset. *M*. (*M*.) *coryli* is widely known as a cosmopolitan species. However, our results suggested that this species might be a species complex.

*Myzocallis* (*Myzocallis*) *boerneri* specimens collected from New Zealand and Italy showed genetic divergence of 8.4% (Fig 14). This species was originally described from Europe and introduced into New Zealand [65]. Assuming species identification of Italian *M*. (*M*.) *boerneri* is correct. Our results suggested that European and New Zealand populations are distinct species.

**Fig 15:**
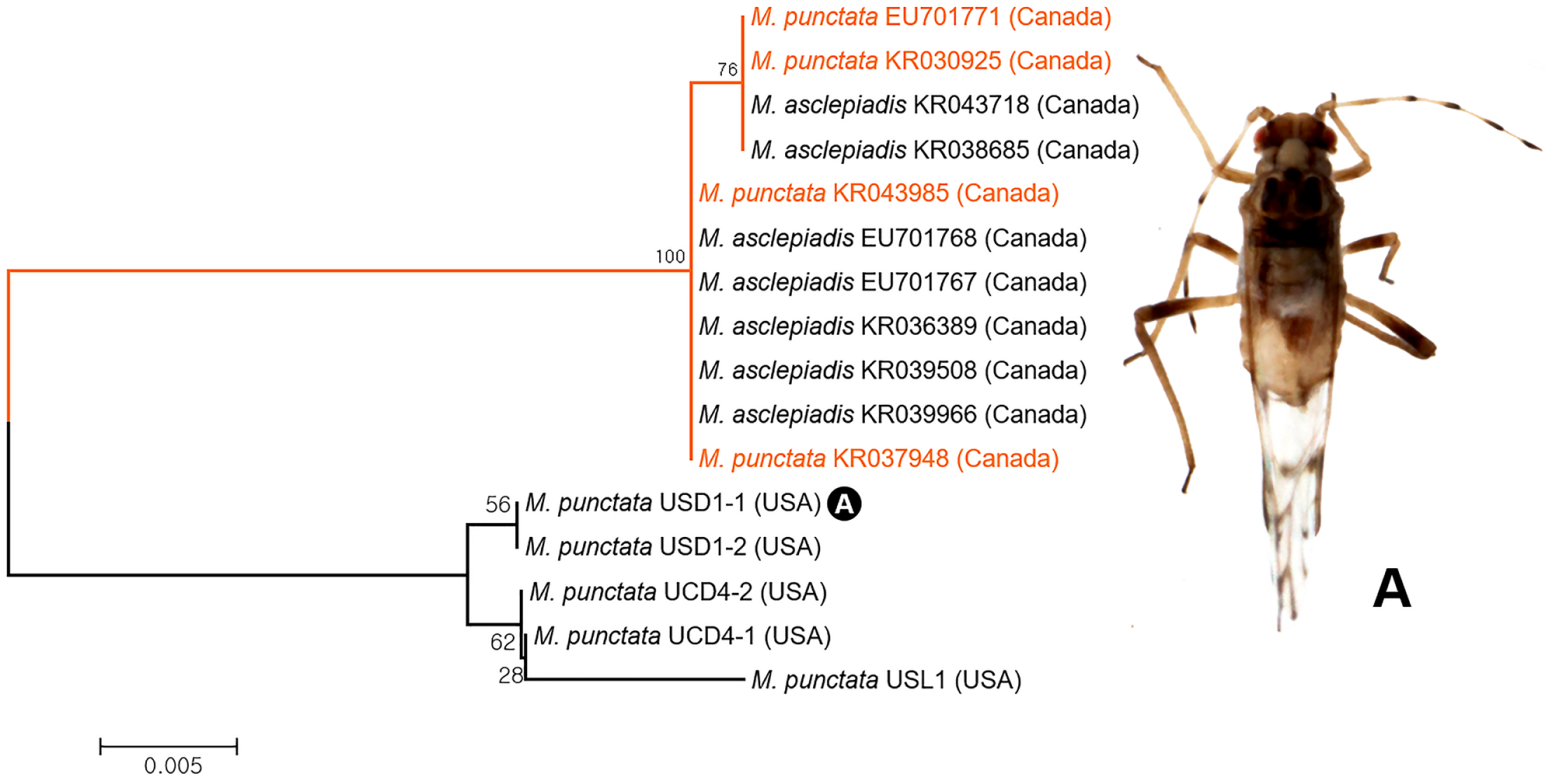
Neighbor-joining tree of *COI* partial gene sequences of subgenus *Neomyzocallis* spp. (16 sequences of 2 morphospecies).

*COI* sequences of two *Neosymydobius* species, *Myzocallis* (*Neosymydobius*) *asclepiadis and M*. (*N*.) *punctata*, revealed possible misidentified sequences of *M*. (*N*.) *punctata* (Fig 15). As a result of ABGD and bPTP analyses, *M*. (*N*.) *punctata* was subdivided into two subgroups (Fig 3). Four sequences of *M*. (*N*.) *punctata* (Genbank accession no. EU701771, KR030925, KR043984, and KR037948) were completely identical to those of *M*. (*N*.) *asclepiadis* rather than to *M*. (*N*.) *punctata* with 100% support value (Fig 15). Our results suggested that 4 individuals (Genbank accession no. EU701771, KR030925, KR043984, and KR037948) of *M*. (*N*.) *punctata* might be misidentification of *M*. (*N*.) *asclepiadis*.

**Fig 16:**
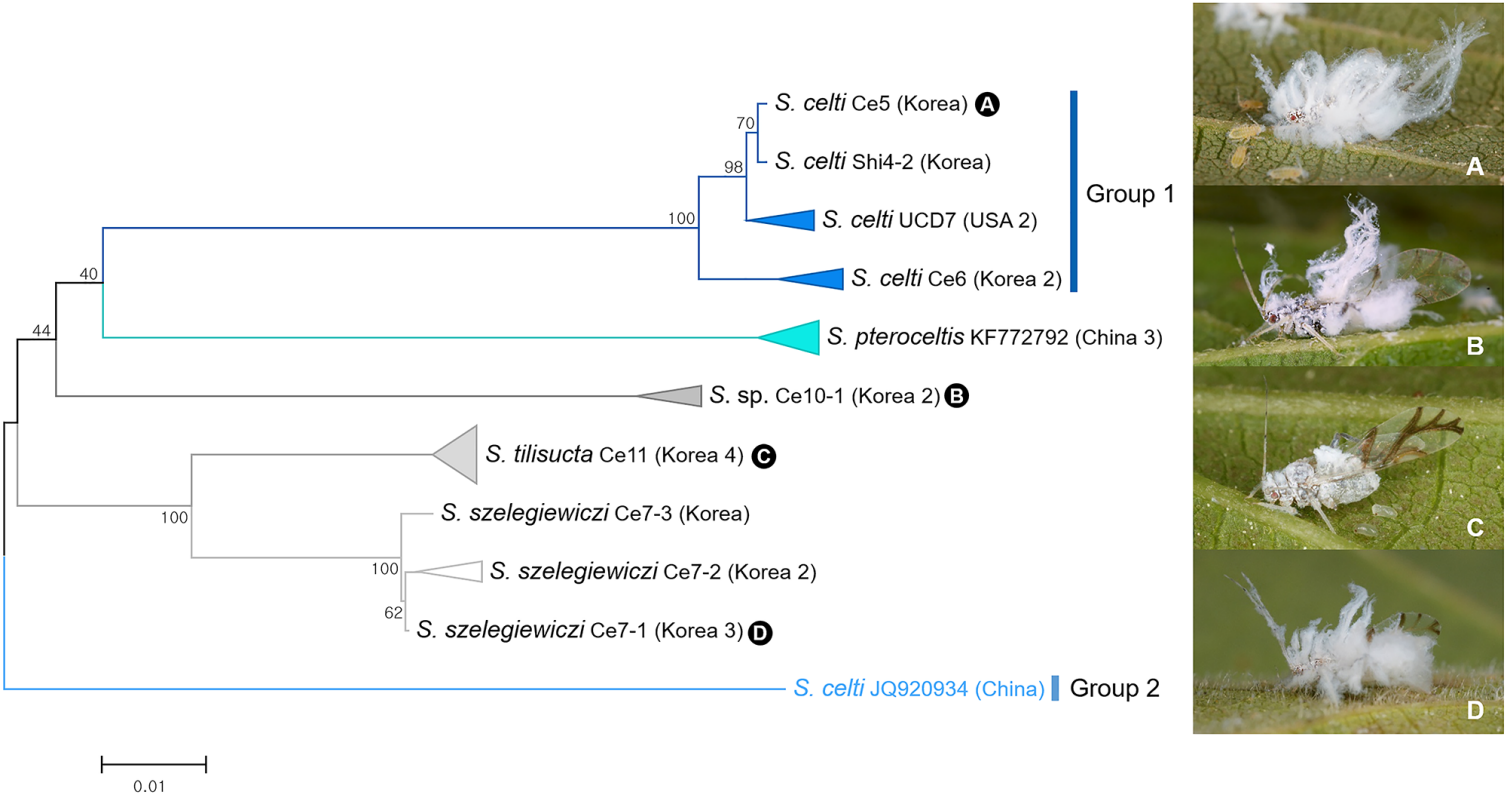
Neighbor-joining tree of *COI* partial gene sequences of *Shivaphis* spp. (22 sequences of 5 morphospecies).

Among 22 specimens of five *Shivaphis* spp., 7 individuals of *Shivaphis celti* showed extremely high levels of intraspecific distance (16.1% to 16.6%) due to a single sequence (Genbank accession no. JQ920934) (Fig 16). Such a high intraspecific distance suggests that this specimen might be a misidentification or a cryptic species. Subsequent morphological re-examination of the voucher specimen for this sequence (Genbank accession no. JQ920934) is needed.

**Fig 17:**
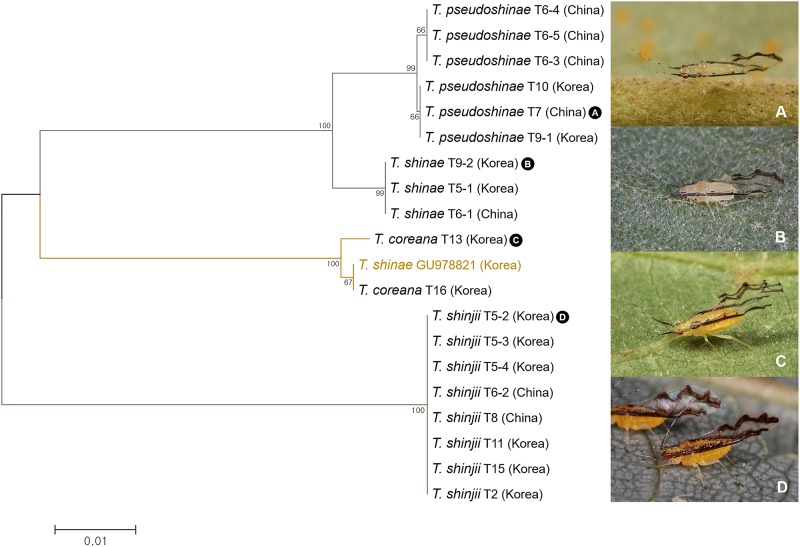
Neighbor-joining tree of *COI* partial gene sequences of *Tiliaphis* spp. (20 sequences of 4 morphospecies).

A total of 20 specimens of four *Tiliaphis* species were analyzed. We found a possible misidentified sequence which was identified as *Tiliaphis shinae* (Genbank accession no. GU978821) (Fig 17). Subsequently, we re-examined the voucher specimen of this sequence and found that this specimen was in fact *T*. *coreana*.

**Fig 18:**
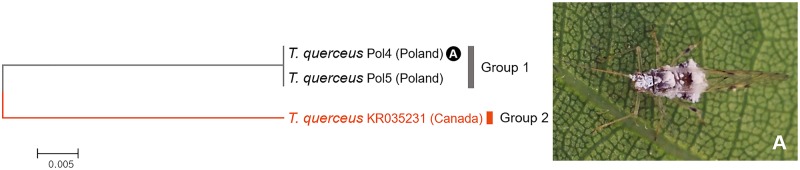
Neighbor-joining tree of *COI* partial gene sequences of *Tuberculatus querceus* (3 sequences).

Three sequences of *Tuberculatus* (*Tuberculatus*) *querceus* were divided into two subgroups with divergence of 8.2% (Fig 18). This species is distributed through Europe to South-western Asia. It is also introduced into Canada [30, 38]. However, our results suggested that European and Canadian specimens might be distinct species.

#### Case III: Low genetic distances between morphologically distinct species

**Fig 19:**
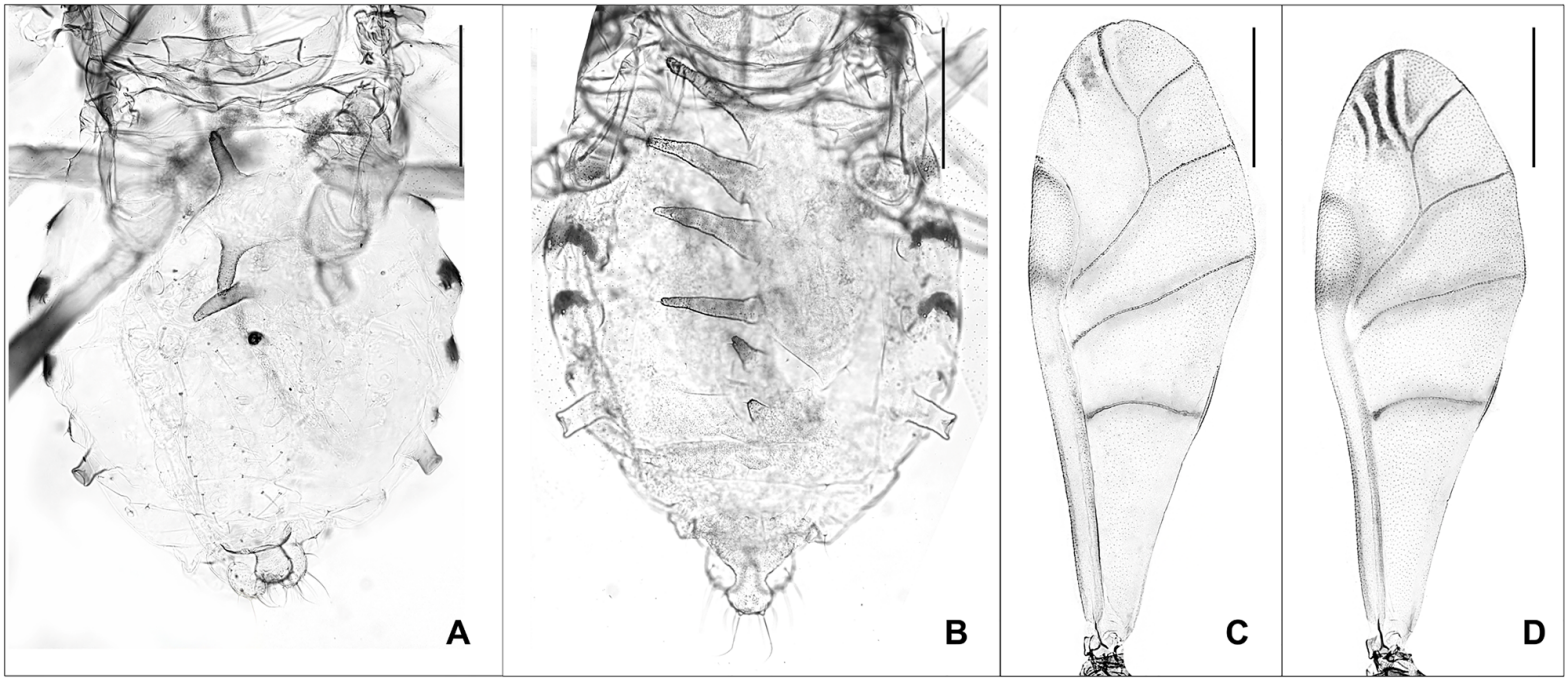
Alate vivipara of *Pterocallis alnijapoinicae* (A, C) and *P*. *nigrostriata* (B, D). (A-B) abdomen. (C-D) forewing (scale bars 0.5mm).

**Fig 20:**
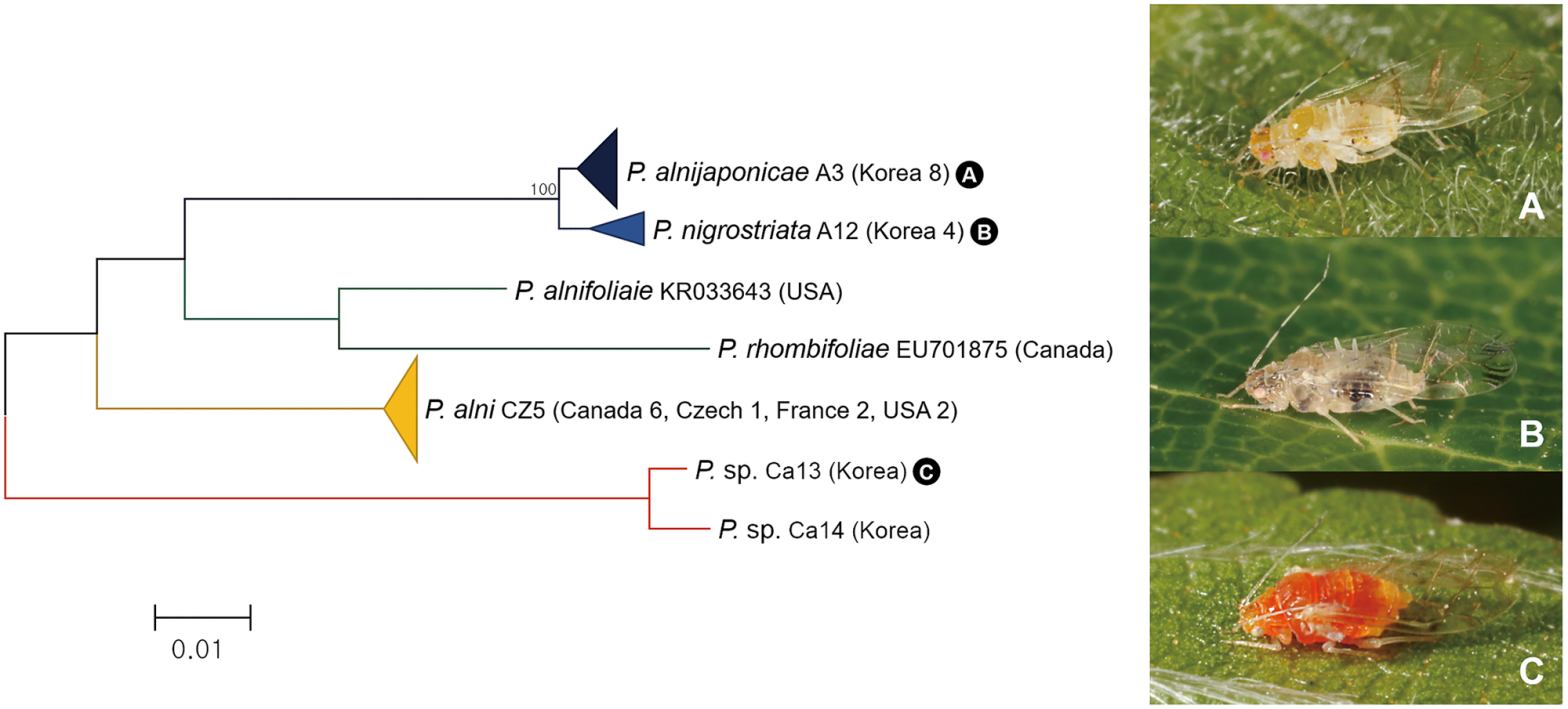
Neighbor-joining tree of *COI* partial gene sequences of *Pterocallis* spp. (27 sequences of 6 morphospecies).

Morphologically, *Pterocallis alnijaponicae* and *P*. *nigrostriata* are easily distinguishable species (Fig 19). As shown in Fig 19, *P*. *nigrostriata* has 5–6 long and conspicuous dorsal abdominal tubercles and forewing with unique marginal patch while *P*. *alnijaponicae* has 3–4 short dorsal abdominal tubercles and forewing without marginal patches. Unexpectedly, low genetic divergence (0.5%) between *P*. *alnijaponicae* and *P*. *nigrostriata* was found (Fig 20). ABGD and bPTP analyses also supported these results by combining *P*. *alnijaponicae* and *P*. *nigrostriata* as a single MOTU (Fig 3). Such discrepancies between morphological and DNA barcoding results raise a question about the validity of these species.

**Fig 21:**
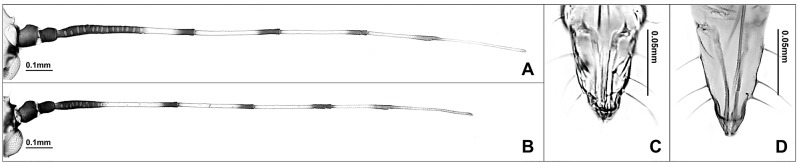
Alate vivipara of *Tiliaphis pseudoshinae* (A, C) and *T*. *shinae* (B, D). (A-B) antenna. (C-D) ultimate rostral segment (scale bars 0.1mm).

*Tiliaphis pseudoshinae* and *T*. *shinae* are morphologically similar. However, *T*. *pseudoshinae* can be distinguished from *T*. *shinae* by having longer ultimate rostral segment, shorter antenna, and less secondary sensoria on the 3rd antennal segment (Fig 21). Between these two species, sequence divergence of 2.1% to 2.5% was observed (Fig 17). Based on ABGD analysis, *T*. *pseudoshinae* and *T*. *shinae* were separated into distinct MOTUs. However, bPTP analysis grouped these two species as a single MOTU (Fig 3). Thus, comparing more specimens with multiple generic markers is needed for better delimitation of these species.

**Fig 22:**
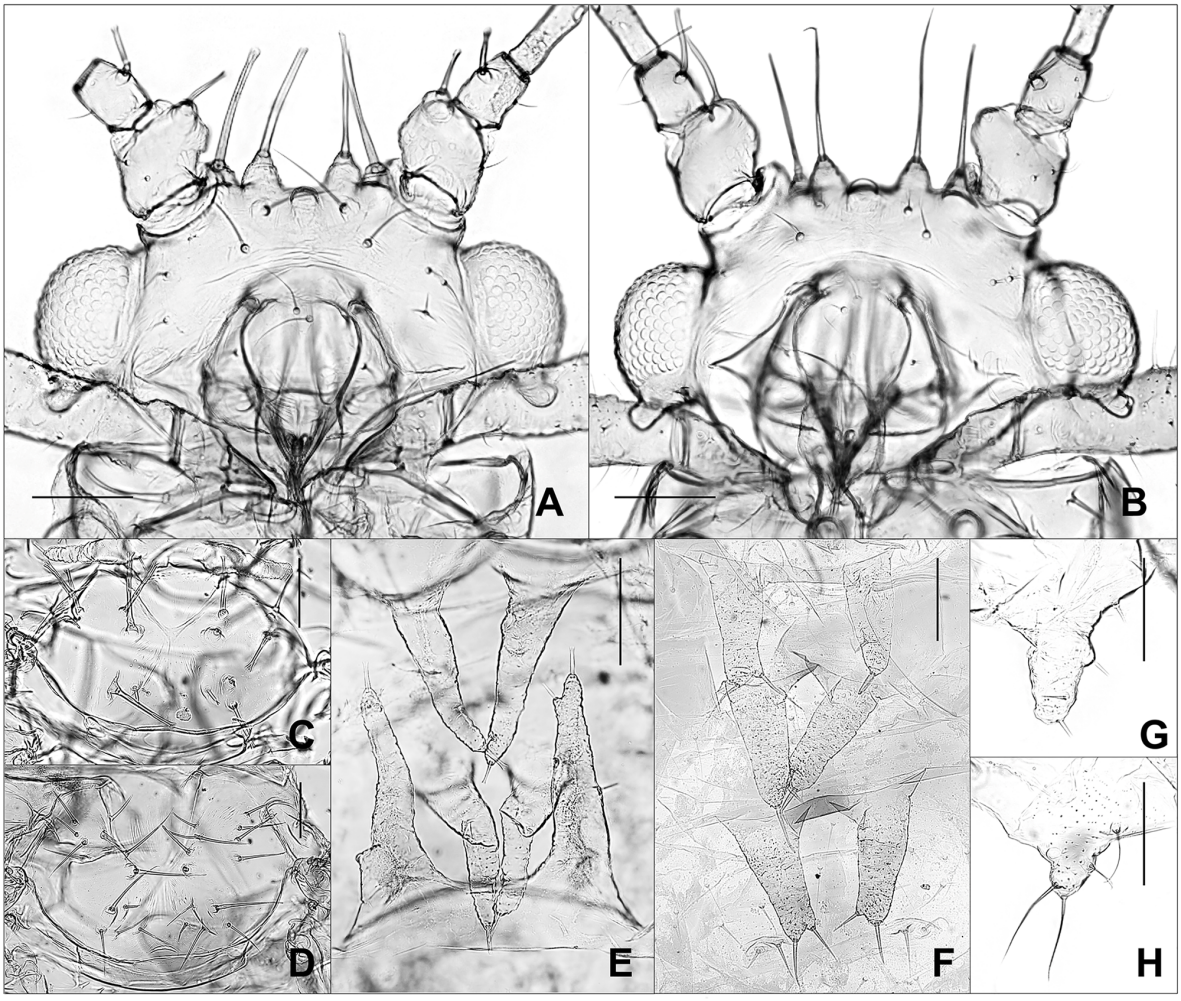
Alate vivipara of *Tuberculaphis* (*Orientuberculoides*) *capitatus* (A, C, E, G) and *T*. (*O*.) *fangi* (B, D, F, H). (A-B) head. (C-D) setae on thorax. (E-F) abdominal dorsal tubercles. (G-H) 4th abdominal marginal tubercle (scale bars, 0.1mm).

Four individuals of *Tuberculatus* (*Orientuberculoides*) *capitatus* and nine individuals of *T*. (*O*.) *fangi* were analyzed. *T*. (*O*.) *capitatus* and to *T*. (*O*.) *fangi* can be distinguished by having different shapes and lengths of setae (Fig 22). However, their genetic divergence was only 0.5% to 1.5% (Fig 12). In fact, different shapes, arrangement, and lengths of hairs are often can be critical characteristics for aphid species delimitation. A review of the variations within and between these species is still required.

#### Case IV: Ambiguous genetic distances

**Fig 23:**
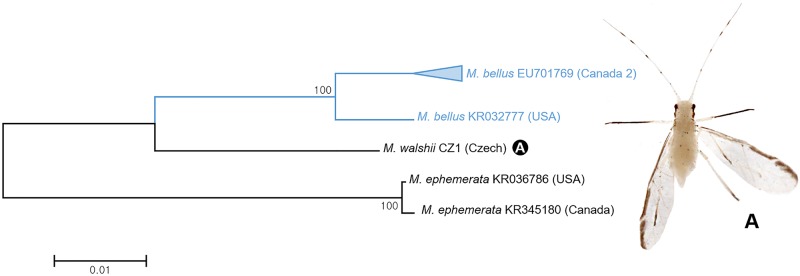
Neighbor-joining tree of *COI* partial gene sequences of subgenus *Lineomyzocallis* spp. (6 sequences).

In this study, six individuals of three species in subgenus *Lineomyzocallis* belonging to genus *Myzocallis* were analyzed. DNA barcoding showed 1.7% intraspecific distance between two Canadian and one American specimen of *Myzocallis* (*L*.) *bellus* (Fig 23). ABGD analysis detected them as a single MOTU. However, bPTP analysis separated these Canadian and American specimens as distinct MOTUs. This situation requires further morphological and molecular analysis.

**Fig 24:**
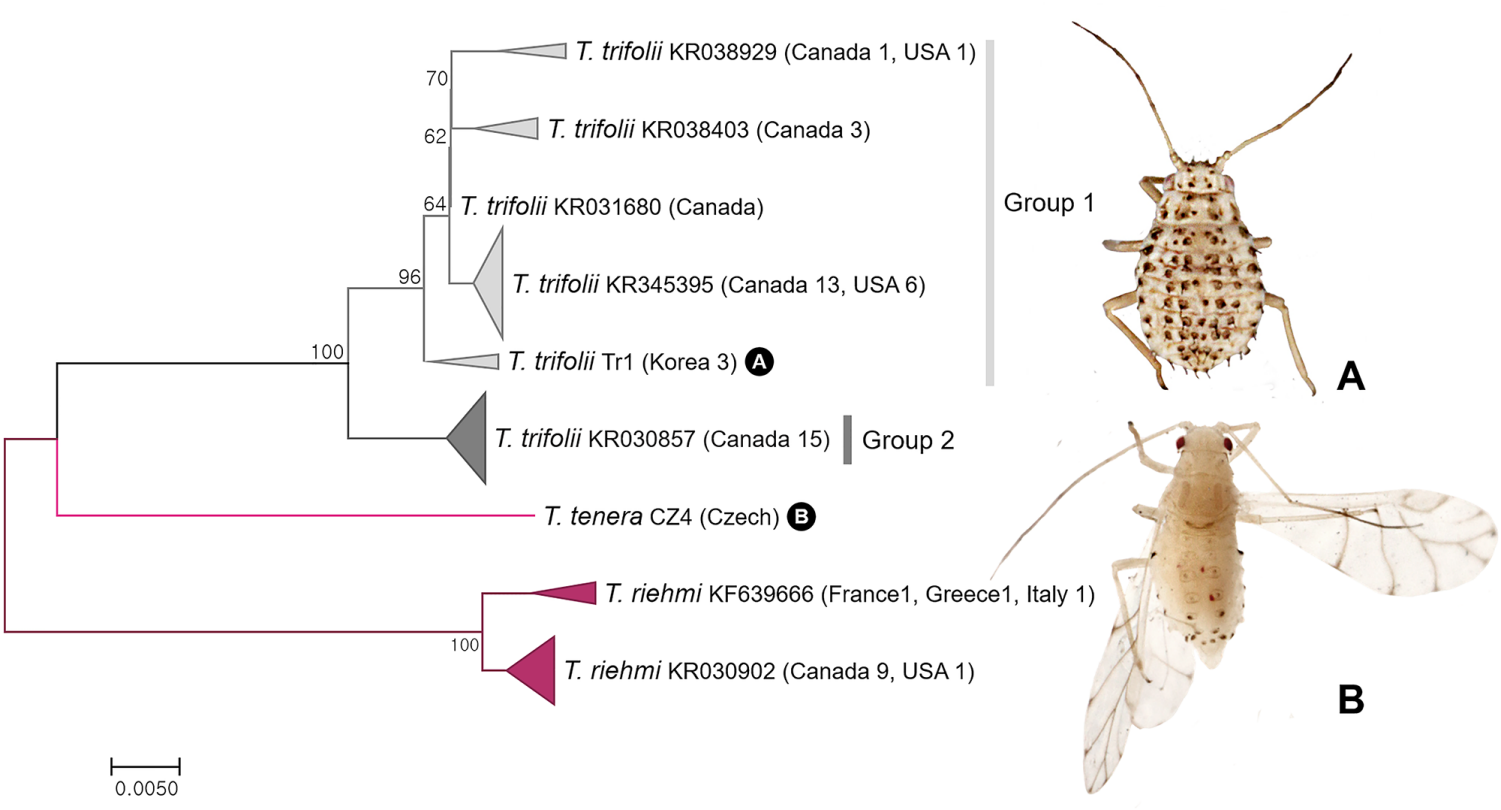
Neighbor-joining tree of *COI* partial gene sequences of genus *Therioaphis* spp. (57 sequences of 3 morphospecies).

A total of 57 specimens belonging to 3 species of *Therioaphis* were analyzed in this study. As shown in Fig 24, 43 specimens of *Therioaphis* (*Pterocallidium*) *trifolii* were subdivided into two subgroups with intergroup barcode divergence of 1.9% (Fig 24). Group 1 comprised of American, Canadian, and Korean specimens while group 2 only contained Canadian specimens. However, both groups formed a single MOTU according to both ABGD and bPTP analyses. There are four subspecies, *T*. (*P*.) *trifolii albae*, *T*. (*P*.) *trifolii maculata*, *T*. (*P*.) *trifolii trifolii* and *T*. (*P*.) *trifolii ventromaculata* in of *T*. (*P*.) *trifolii* [14, 38]. Detailed molecular studies have not been conducted on this group of taxa. Thus, comparing worldwide samples of *T*. (*P*.) *trifolii* spp. is needed in future studies.

**Fig 25:**
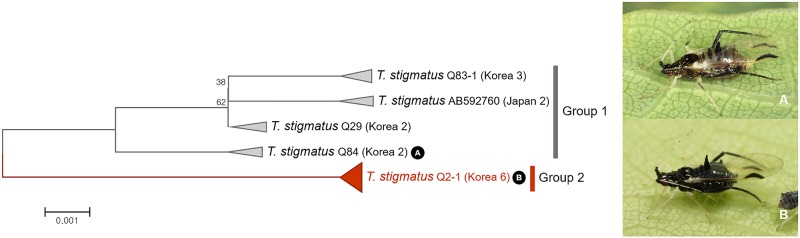
Neighbor-joining tree of *COI* partial gene sequences of *Tuberculatus* (*Arakawana*) *stigmatus* (15 sequences).

Within *Tuberculatus* (*Arakawana*) *stigmatus*, two distinct subgroups were detected based on NJ tree and bPTP analyses with genetic divergence of 1.5% to 1.9% (Figs 3 and 25). Individuals in group 1 and group 2 showed distinct morphological features (S10 Fig). Group 2 has shorter siphunculi and small abdominal marginal tubercles without marginal tubercle on the 5th abdominal segment (S10 Fig). Based on their morphological differences, group 1 and group 2 might be distinct species despite the relatively low divergence level between these two subgroups.

**Fig 26:**
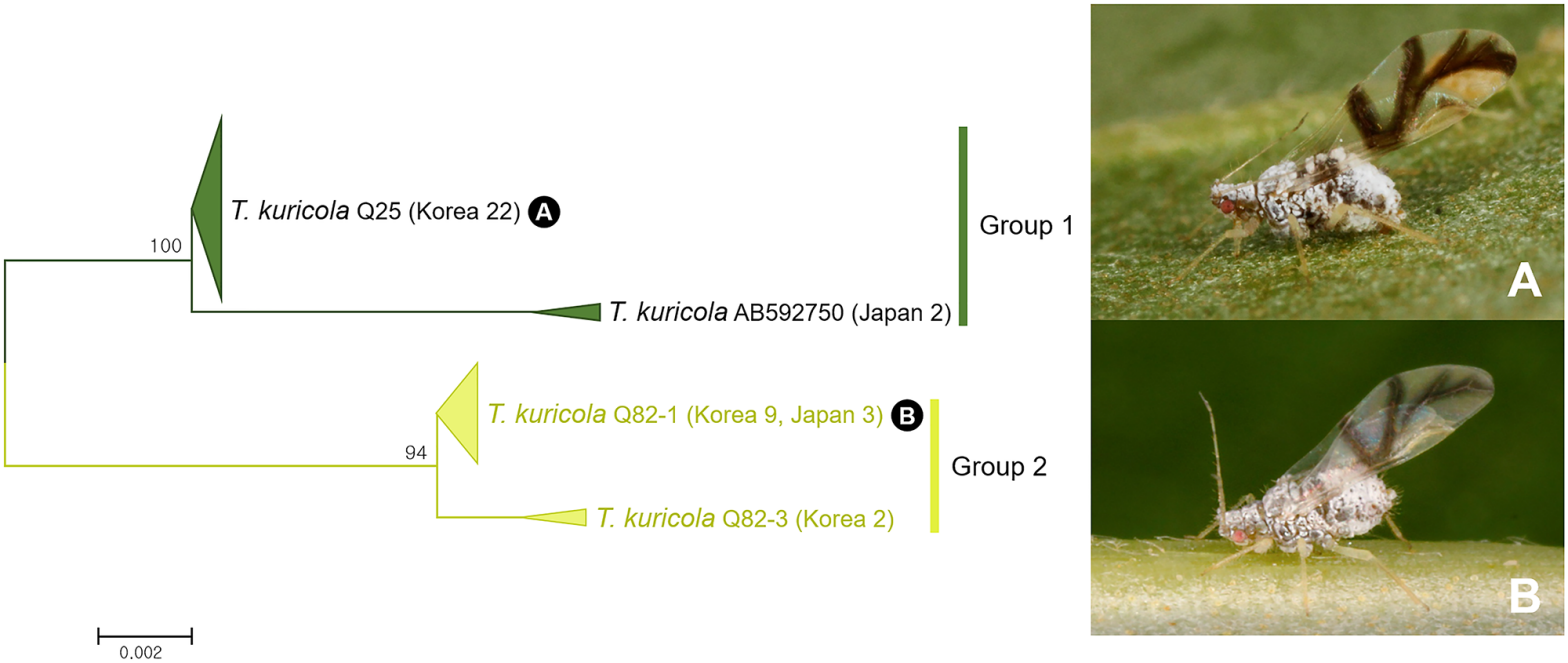
Neighbor-joining tree of *COI* partial gene sequences of *Tuberculatus* (*Nippocallis*) *kuricola* (38 sequences).

Among 38 individuals of *Tuberculatus* (*Nippocallis*) *kuricola*, two subgroups (group 1 and group 2) were detected based on NJ tree and bPTP analysis (Fig 3). Genetic divergence between the two groups was 1.9% (Fig 26). Morphologically, the two groups are superficially similar. However, group 2 can be recognized by having 3–4 setae on each dorsal abdominal tubercle (rather than 2 in group 1) with a shorter ultimate rostral segment (S11 Fig). Takahashi (1936) has described subspecies *T*. (*N*.) *kuricola cantoensis*. However, it is currently unclear whether *T*. (*N*.) *kuricola cantoensis* and *T*. (*N*.) *kuricola kuricola* correspond to these two genetically divided subgroups.

## Discussion

This study demonstrates that DNA barcoding can be used to reliably identify aphid species in the subfamily Calaphidinae. DNA barcoding of 115 morphospecies (899 sequences) revealed 25 discordances between DNA barcoding results and morphology results. These conflicts involved 18 cases of exceptionally high intraspecific distances, three morphologically distinct species pairs with low genetic distances and four cases of ambiguous intraspecific distances. Except for four undeterminable cases, a total of 15 cryptic species were identified from 12 morphospecies. Among these cases, slight morphological differences were detected in seven species complexes. In the most cases, morphological differences were due to different lengths of various body parts rather than a different shapes or numerical characters. Slight length differences are easy to be overlooked. They can be obscured by intraspecific variations. From these results, we can infer the presence of cryptic diversity in Calaphidinae.

In this study, distinct host-plant associations were revealed in three species complexes: *Mesocallis corylicola*, *Tinocallis* (*Tinocallis*) *zelkowae* and *Tuberculatus* (*Acanthocallis*) *quercicola*. Differences in host-plant associations are often critical for aphid species identification. Host-plant shifts and subsequent genetic differentiation can lead to speciation (ecological species concept) [66–69]. These ecological differences between cryptic species emphasize the necessity to reexamine different host-plant associated populations in a single species.

We also confirmed that species with a wide distribution could have more possibilities to include cryptic species. Some species assessed in this study demonstrated this assumption. For example, *Calaphis flava*, which has been known as invasive species with cosmopolitan distribution, included subgroups with each restricted collection area. However, most previous DNA barcoding studies in aphids targeted samples only collected within restricted area [7, 35, 64]. Our results suggest that multiregional sampling would be very important to recognize cryptic diversity.

However, we also found deep intraspecific divergence within species complexes without obvious host-plant and geographic difference. In fact, groups within *Takecallis arundicolens* and *Tuberculatus* (*Nippocallis*) *kuricola* were found together within the same individual colony, respectively. In particular, viviparous females of *T*. *arundicolens* (group 1 and group 3) were not distinguishable by morphology. Considering that both bamboo and chestnut trees are urban landscape plants, the phenomenon (genetically distinct groups occur in the same colony) might indicate that human mediated transportation might have played a role in subsequent colony merging.

We found three morphologically distinct species pairs with relatively low genetic distances: *Pterocallis alnijaponicae* and *P*. *nigrostriata*, *Tiliaphis pseudoshinae* and *T*. *shinae* and *Tuberculatus* (*Orientuberculoides*) *capitatus* and *T*. (*O*.) *fangi*. This could be due to many reasons such as rapid radiation, balanced polymorphism and introgressive hybridization [70]. In the three DNA barcode sharing species pairs, it was common that each pair of species was collected together on the same host-plants and geographical regions. Considering that, there can be opportunities of hybridization between species pair during sexual reproduction on the same host-plant. Such phenomenon has been frequently reported in several taxa of aphids [71–73]. However, conducting additional ecological and molecular research is needed to verify whether barcode sharing is due to hybridization.

In insects, species delimitation threshold value from 2 to 5% of divergence, depending on the group, is generally accepted as a means to estimate species boundaries. For example, 2% intraspecific divergence may indicate the existence of hidden species in Lepidoptera [11, 74]. However, within Diptera, this level of divergence only represents an intraspecific difference [75–77]. In the suborder Heteroptera, threshold value of 2.2% has been applied for DNA barcode based species delimitation [78–79]. In this study, about 2.5% of species delimitation value could be applied for most of species. However, there is clear limits applying certain species delimitation threshold value in every case. In this study, we found highly varied interspecific genetic variation for *COI*, from 0.5% to 20.1% between congeneric species of Calaphidinae. These results suggested that applying a single threshold value might not work for all members in this group. To get more accurate species delimitation results, combination of additional information such as different genetic markers, morphological characters, and ecological differences is required. For example, in the present study, morphological re-examination of two species, *Tuberculatus* (*Arakawana*) *stigmatus* and *Tuberculatus* (*Nippocallis*) *kuricola*, with intraspecific divergence of 1.9% resulted in the detection of morphological differences, suggesting that additional species are present.

Some aphid species are important pest of various crop and ornamental plants. They play a critical role in ecosystems. Although DNA barcoding has been carried out several times for aphids before, most studies have been mainly focused on the largest subfamily Aphidinae with less emphasis on other subfamilies [7, 13, 30, 64]. DNA barcodes produced in this study are of value in aiding in the identification of species of Calaphidinae. Further, remarkable cryptic diversity and suspicious cases such as barcode sharing species pairs are detected in this study. Our findings suggest that many more cryptic diversity are not yet been uncovered in aphids. For more accurate and higher resolution of possibly overlooked species diversity investigation, future studies should focus on well-designed sampling plan to reflect morphological, ecological and distributional diversities within species.

## Supporting information

S1 Fig Neighbor-joinig tree for the 899 individuals of 115 morphospecies based on *COI* barcoding region. (TIF)

S2 Fig Alate vivipara of 2 subgroups of *Eucallipterus tiliae* group 1 (A-B) and group 2 (C-D). (A, C) antenna. (B, D) 2nd segment of hind tarsi (scale bars, 0.1mm).
(TIF)

S3 Fig Alate vivipara of 2 subgroups of *Mesocallis corylicola* group 1 (A, C) and group 2 (B, D). (A-B) siphunculi. (C-D) ultimate rostral segment (scale bars, 0.05mm).
(TIF)

S4 Fig Alatoid nymph of 3 subgroups of *Takecallis arundicolens* group 1 (A), group 2 (B) and group 3 (C). (A-C) body (scale bars, 0.5mm).
(TIF)

S5 Fig Alate vivipara of 2 subgroups of *Tinocallis zelkowae* group 1 (A, C) and group 2 (B, D). (A-B) ultimate rostral segment. (C-D) abdomen.
(TIF)

S6 Fig Alate vivipara of 2 subgroups of *Tuberculatus* (*Acanthocallis*) *quercicola* group 2 (A) and group 3 (B). (A-B) antenna (scale bars 0.1mm).
(TIF)

S7 Fig Alate vivipara of 4 subgroups of *Tuberculatus* (*Orientuberculoides*) *higuchii* group 1 (A, E, I, M), group 2 (B, F, J, N), group 3 (C, G, K, O) and group 4 (D, H, L, P). (A-D) cauda. (E-H) siphunculi. (I-L) 2nd segment of hind tarsi. (M-P) antenna (scale bars 0.1mm).
(TIF)

S8 Fig Alate vivipara of 2 subgroups of *Tuberculatus* (*Orientuberculoides*) *yokoyamai* group 1 (A, C, E) and group 2 (B, D, F). (A-B) antanna. (C-D) cauda. (E-F) siphunculi (scale bars 0.1mm).
(TIF)

S9 Fig Alate vivipara of 2 subgroups of *Tuberculatus* (*Tuberculoides*) *annulatus* group 1 (A, C) and group 2 (B, D). (A-B) antenna. (C-D) cauda (scale bars, 0.1mm).
(TIF)

S10 Fig Alate vivipara of 2 subgroups of *Tuberculatus* (*Arakawana*) *stigmatus*, group 1 (A, C, E) and group 2 (B, D, F). (A-B) abdomen. (C-D) siphunculi. (E-F) antenna.
(TIF)

S11 Fig Alate vivipara of 2 subgroups of *Tuberculatus* (*Nippocallis*) *kuricola*, group 1 (A, C) and group 2 (B, D). (A-B) ultimate rostral segment. (C-D) abdomen.
(TIF)

S1 Table Detailed collection information and Genbank accession numbers of species used in this study. (XLSX)

S2 Table Detailed collection information and Genbank accession numbers of sequences downloaded from Genbank. (XLSX)
