# Supplementary figures and images for: Cryptic diversity of the subfamily Calaphidinae (Hemiptera: Aphididae) revealed by comprehensive DNA barcoding
Source: PLoS One. 2017 Apr 27;12(4):e0176582. doi: 10.1371/journal.pone.0176582 (PMC5407777; doi:10.1371/journal.pone.0176582)

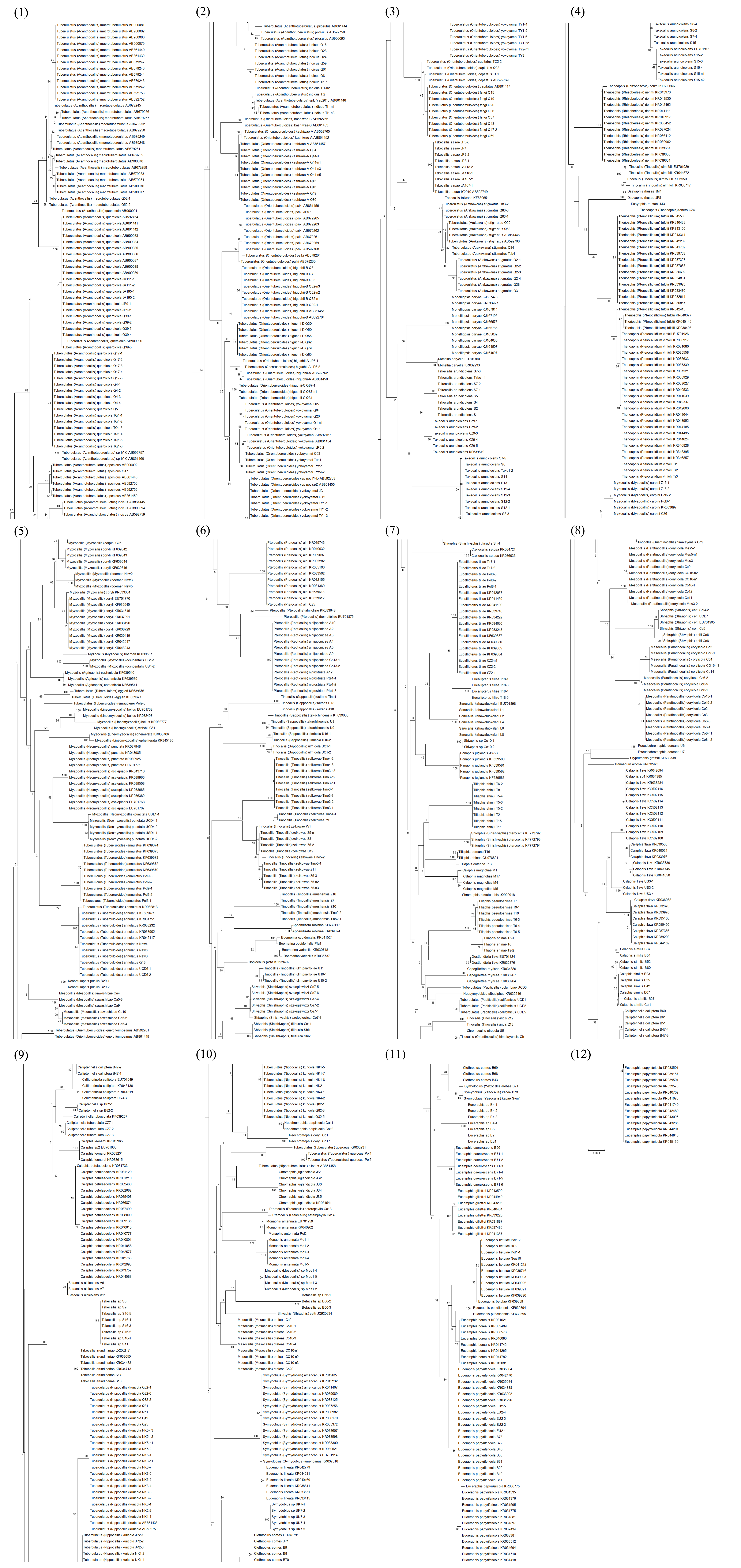

Supplement: S1 Fig — (TIF) [file pone.0176582.s001.tif]

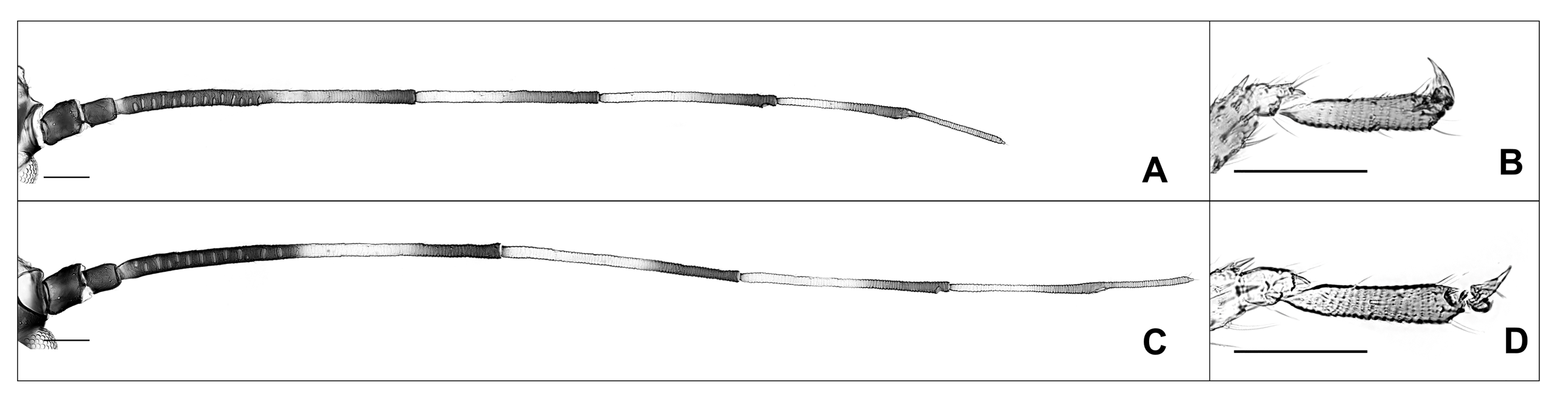

Supplement: S2 Fig — (A, C) antenna. (B, D) 2nd segment of hind tarsi (scale bars, 0.1mm). (TIF) [file pone.0176582.s002.tif]

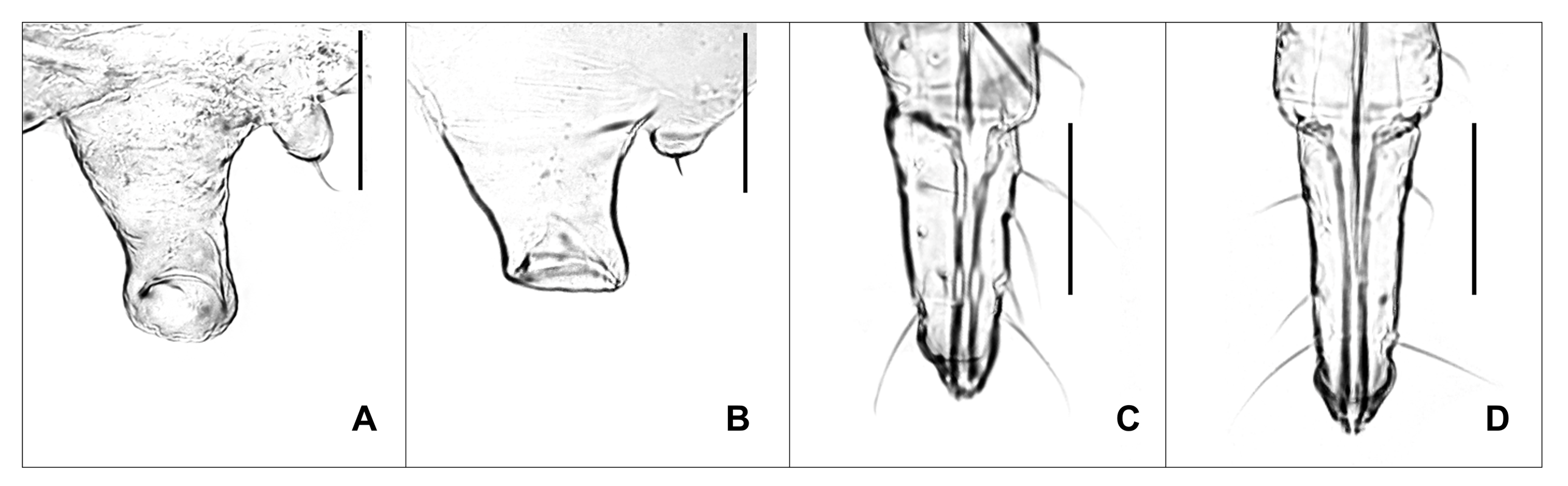

Supplement: S3 Fig — (A-B) siphunculi. (C-D) ultimate rostral segment (scale bars, 0.05mm). (TIF) [file pone.0176582.s003.tif]

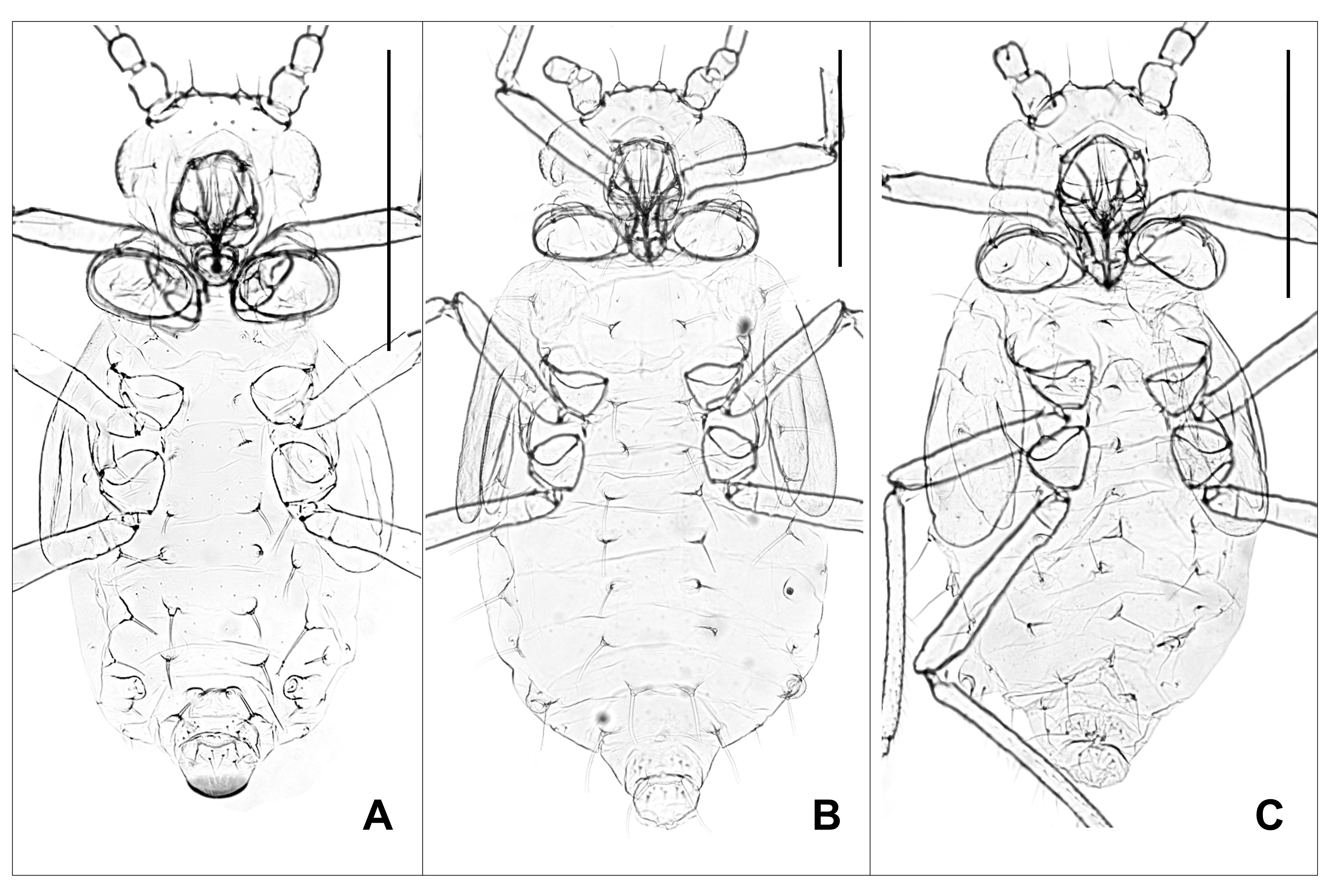

Supplement: S4 Fig — (A-C) body (scale bars, 0.5mm). (TIF) [file pone.0176582.s004.tif]

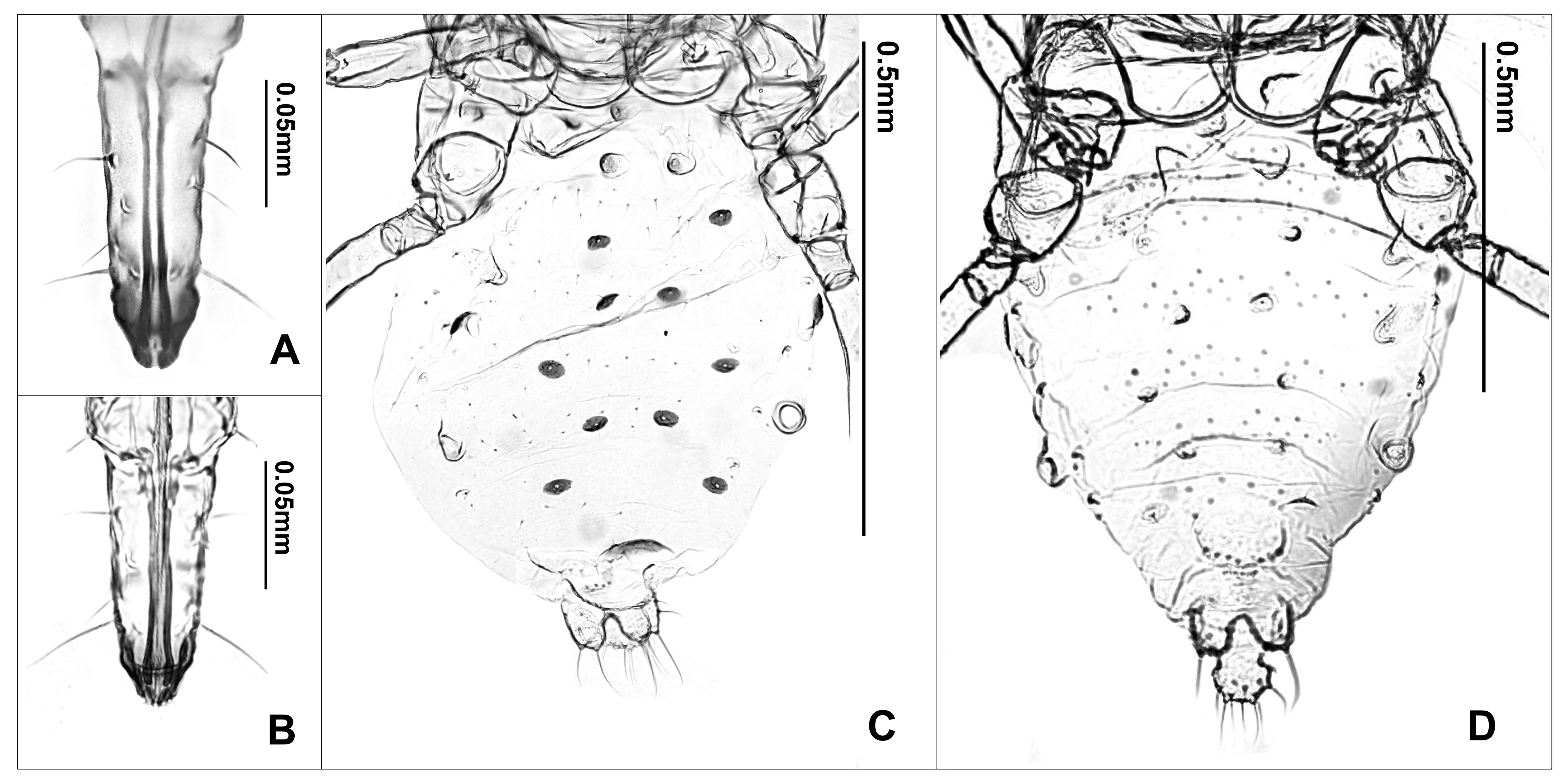

Supplement: S5 Fig — (A-B) ultimate rostral segment. (C-D) abdomen. (TIF) [file pone.0176582.s005.tif]

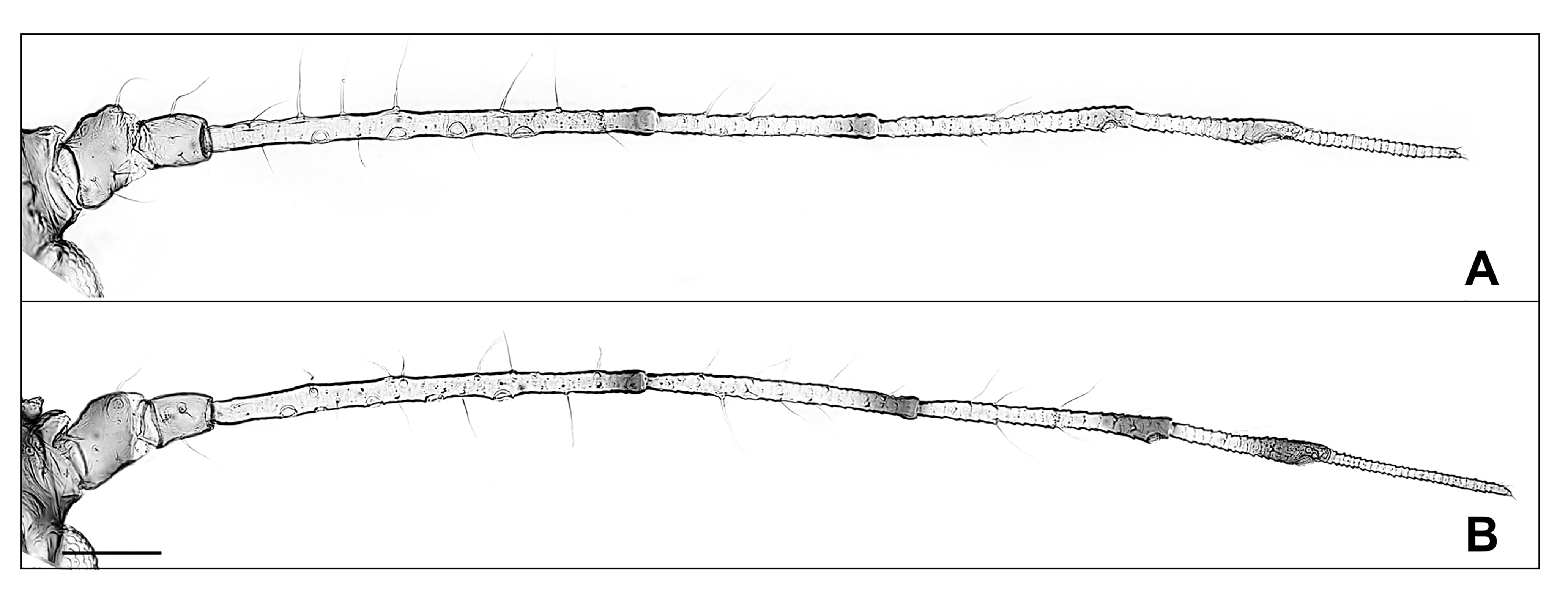

Supplement: S6 Fig — (A-B) antenna (scale bars 0.1mm). (TIF) [file pone.0176582.s006.tif]

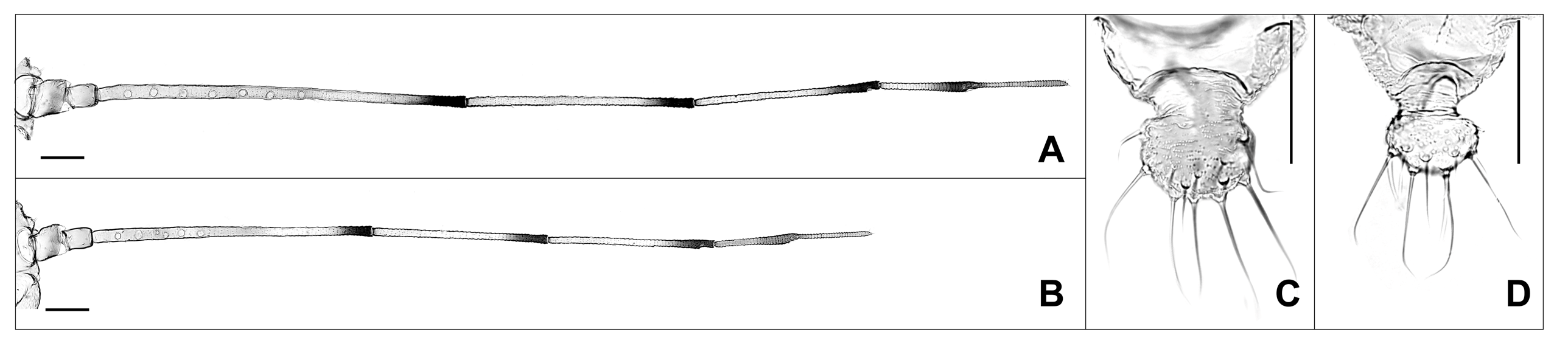

Supplement: S9 Fig — (A-B) antenna. (C-D) cauda (scale bars, 0.1mm). (TIF) [file pone.0176582.s009.tif]

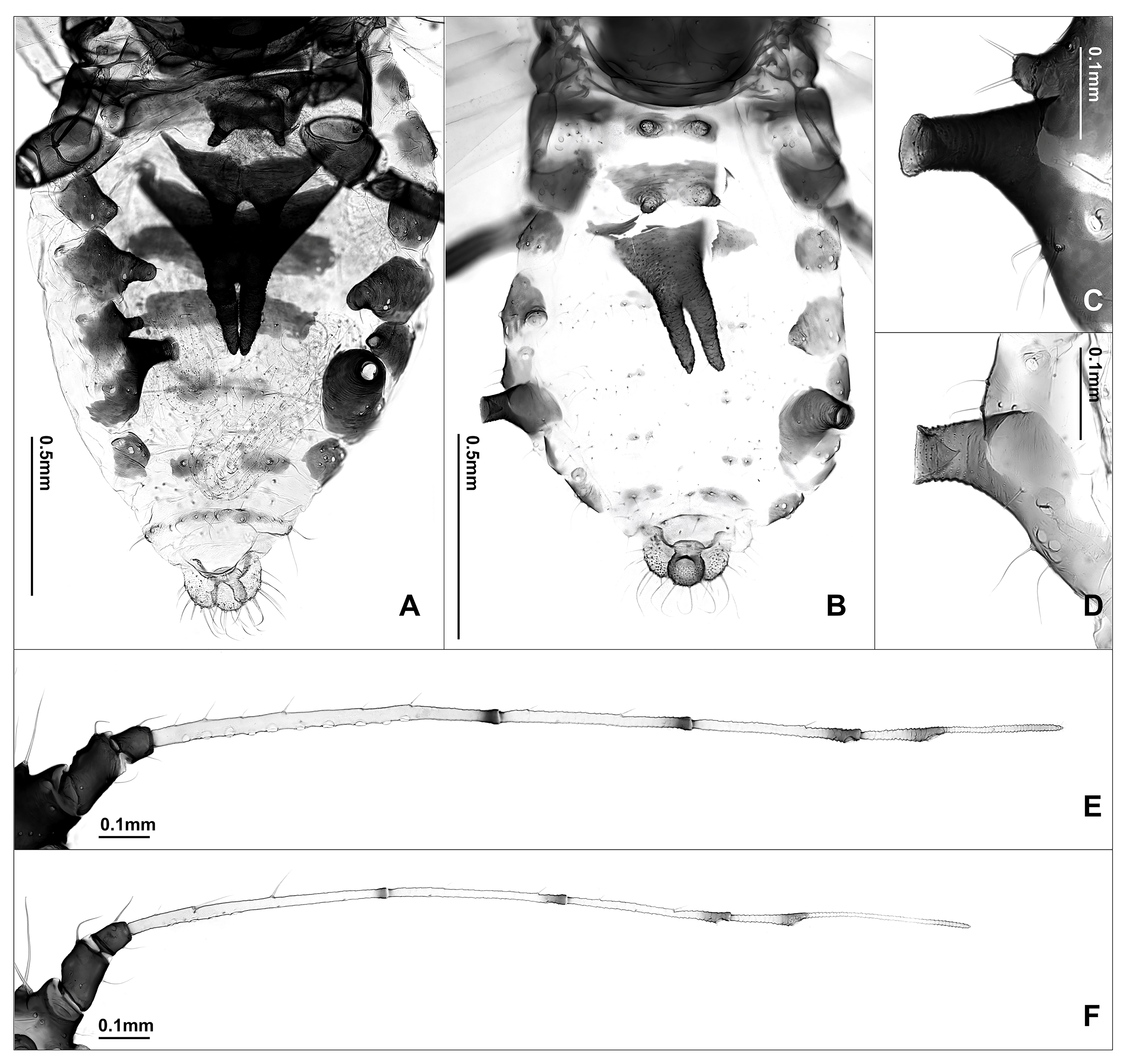

Supplement: S10 Fig — (A-B) abdomen. (C-D) siphunculi. (E-F) antenna. (TIF) [file pone.0176582.s010.tif]

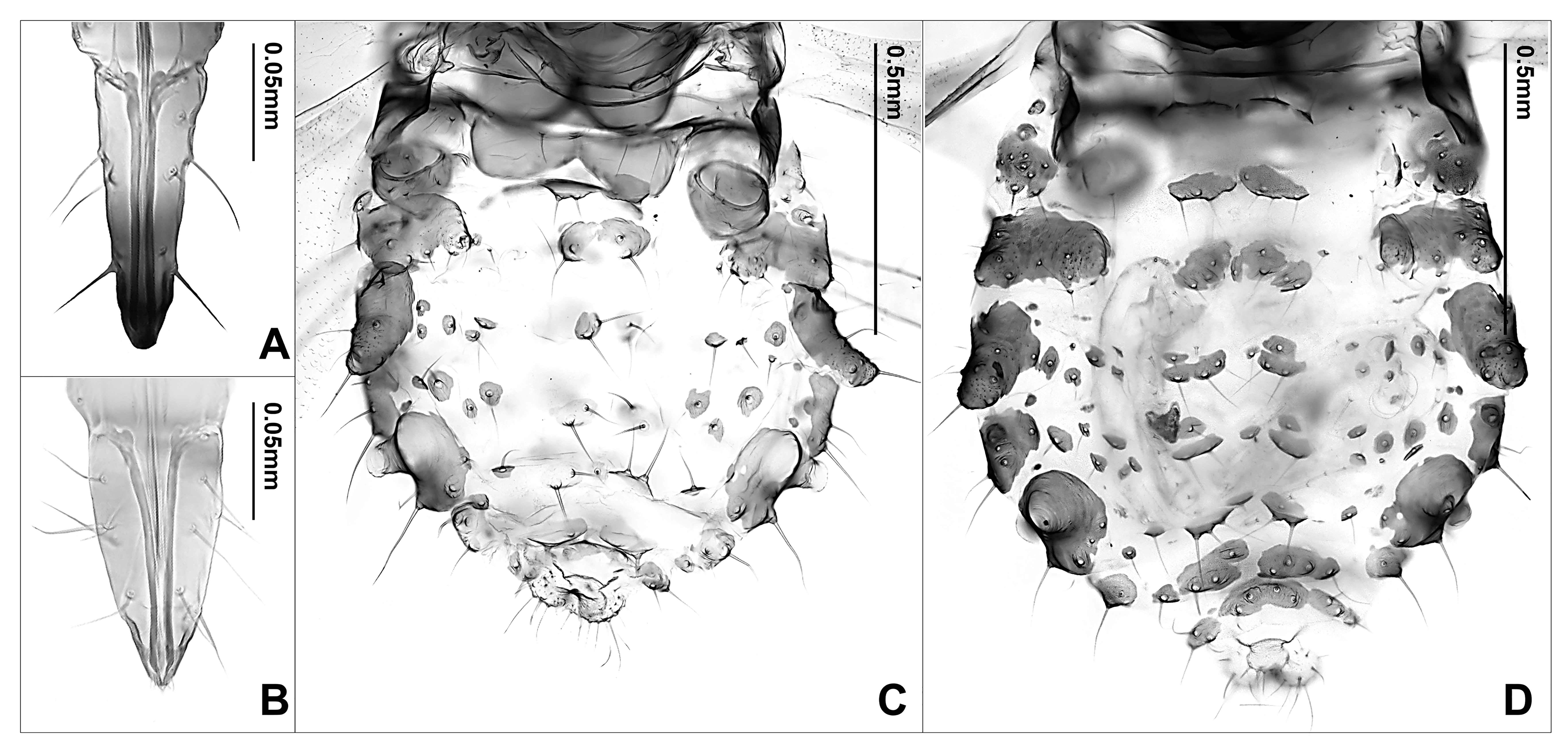

Supplement: S11 Fig — (A-B) ultimate rostral segment. (C-D) abdomen. (TIF) [file pone.0176582.s011.tif]
